# Comparative study of adipose tissue derived mesenchymal stem cells with rapamycin on paraquat-induced acute lung injury and pulmonary fibrosis in a mouse model: histological and biochemical study

**DOI:** 10.1186/s13287-025-04498-w

**Published:** 2025-07-15

**Authors:** Heba Fikry, Lobna A. Saleh, Doaa R. Sadek

**Affiliations:** 1 Department of Histology and Cell Biology, Faculty of Medicine, Ain Shams University, Khalifa El-Maamon st, Abbasiya sq., Cairo, 11566 Egypt; 2 Department of Clinical Pharmacology, Faculty of Medicine, Ain Shams University, Khalifa El-Maamon st, Abbasiya sq., Cairo, 11566 Egypt

**Keywords:** Mesenchymal stem cells, Adipose tissue, Lung injury, Pulmonary fibrosis, Rapamycin, Paraquat, Anti-inflammatory, Antioxidants, Immunohistochemistry, Electron microscopy

## Abstract

**Background:**

The most noticeable consequence of paraquat (PQ) toxicity is pulmonary fibrosis. Mesenchymal stem cells have the remarkable ability to self-renew and differentiate into many cell types. One such type is adipose tissue-derived Mesenchymal Stem Cells (AT-MSCs), which are derived from adipose tissue. Thus, the purpose of this study was to compare the effects of AT-MSCs and rapamycin on paraquat-induced acute lung injury and pulmonary fibrosis in a mouse model.

**Methods:**

Fifty female mice were randomly allocated to four groups. Group I (control group) received the drug solvent using the same route of administration for the same duration as the corresponding experimental groups. Group II (pulmonary fibrosis group) lung injury was induced by injection of PQ at a dosage of 40 mg/kg. Group III (AT-MSCs group) received 1.0 × 10^5^ cells/mouse of male AT-MSCs. Group IV (rapamycin group) received 2.5 mg/kg/day diluted in 1% Dimethyl sulfoxide orally for two weeks. Lung tissue was harvested at the end of the experiment and analyzed by light and electron microscopy in addition to immunohistochemistry evaluation of p53. Samples were also taken to -80 for identification of the Y chromosome (SRY gene) and biochemical testing in the lung tissue. The injured lung was improved with AT-MSCs. Just like the control group, they restored p53 levels.

**Results:**

Following injection with AT-MSCs, there was an increase in Y-chromosome expression levels. Treatment with AT-MSCs also reduced malondialdehyde levels in the lung tissues while increasing superoxide dismutase and reduced glutathione levels. The results showed that pulmonary fibrosis may be mitigated following AT-MSCs transplantation in PQ-poisoned mice by suppressing the synthesis of pro-inflammatory cytokines (interleukin-6 and tumor necrosis factor-alpha) in the lung tissues of the animals. The SRY gene’s expression was significantly upregulated in the AT-MSCs treatment group.

**Conclusion:**

This study provides more evidence that the immunomodulatory effects of AT-MSC transplantation can alleviate pulmonary inflammatory and fibrotic alterations more effectively than rapamycin. As a result, these cells are a very promising pharmacological therapy for acute lung injury and pulmonary fibrosis induced by PQ poisoning.

**Supplementary Information:**

The online version contains supplementary material available at 10.1186/s13287-025-04498-w.

## Introduction

Pesticide poisoning is a significant issue in public health around the world, especially in developing nations. Extremely harmful to both people and other animals, paraquat (PQ, 1,1-dimethyl-4,4-bipyridinium dichloride) is a leading pesticide in agricultural applications [1]. PQ is a bipyridine quaternary amine herbicide that is very effective, inexpensive, and safe for the environment [2]. The lungs aren’t the only organs that PQ damages; the heart, kidneys, GI tract, neurological system, and immunological system are all affected as well [3]. Acute lung injury (ALI) and subsequent fatal pulmonary fibrosis are the main outcomes of PQ poisoning, which mostly affects the lungs. Many people, particularly in underdeveloped nations, have died from pulmonary fibrosis as a consequence of consuming PQ [4]. For this reason, PQ-induced pulmonary injury in mice has been utilized as an experimental model for lung fibrosis [5].

Alveolar epithelial cells may actively uptake PQ via an intrinsic transport system, resulting in 90% of ingested PQ accumulating in lung tissue, which is up to 10-fold more than its concentration in plasma [6]. After damaging the alveolar epithelium, accumulated PQ causes inflammatory cell stimulation, fibroblast activation, excessive extracellular matrix (ECM) formation, and accumulation, with gradual and irreversible lung fibrogenesis [7].

Pulmonary fibrosis and respiratory failure can result from oxidative stress caused by PQ, which is known to generate reactive oxygen species (ROS). This stress also causes disruptions of alveolar epithelial cells, inflammatory cell infiltration into the interstitial and alveolar spaces, proliferation of fibroblasts, and excessive deposition of collagen [8] and inflammatory cytokines release (tumor necrosis factor-α (TNF-α) and transforming growth factor-β (TGF-β) [9].

Idiopathic pulmonary fibrosis (IPF) is the most prevalent form of pulmonary fibrosis. It is defined as a chronic, progressive, and irreversible interstitial pneumonia that is brought about by an overabundance of extracellular matrix components, such as activated fibroblasts and myofibroblasts expressing α-smooth muscle actin (α-SMA) [10–12]. Unfortunately, many patients are resistant to various treatments such as antioxidants and immunosuppressants. This highlights the critical need for immediate research into novel therapeutic approaches for pulmonary fibrosis.

Owing to the limitations of the modeling cycle and the diverse mechanisms associated with PQ-induced oxidative stress, including inflammation and lipid peroxidation, the majority of antioxidants administered to animals with PQ-induced ALI and pulmonary fibrosis failed to concurrently mitigate the toxicity of both conditions. Therefore, it would be beneficial to treat PQ poisoning with a medication that has antioxidant and anti-inflammatory characteristics [7, 13, 14].

Rapamycin (sirolimus) is a lipophilic antibiotic sourced from Streptomyces hygroscopicus and is classified within the macrolide family, possessing immunosuppressive effects comparable to ciclosporin and tacrolimus [15]. Although the specific mechanism is yet unknown, rapamycin has the potential to help treat pulmonary fibrosis and acute lung injury induced by PQ [16]. One mechanism by which rapamycin protects against oxidative stress is by increasing the Nuclear factor E2-related factor 2 (Nrf2) pathway-mediated transcription of antioxidant genes [17]. Rapamycin has a clinically potent anti-fibrotic role [18].

The great accessibility, low invasiveness, and lack of ethical constraints of adipose tissue-derived mesenchymal stem cells (AT-MSCs) make them a promising candidate among mesenchymal stem cells (MSCs) [19]. Significantly, adipose tissue possesses a higher density of MSCs than bone marrow. Aside from their diversity, AT-MSCs outperform BM-MSCs in terms of growth factors produced, immunomodulatory capabilities, and differentiation potential when it comes to repairing and regenerating damaged tissues [19]. Over time, AT-MSCs have evolved into the “seed cell” for studies on stem cell treatment for ischemic heart failure.

Therefore, this research aimed to compare the effects of adipose tissue-derived Mesenchymal Stem Cells and rapamycin in a murine model of paraquat-induced acute lung injury and pulmonary fibrosis.

## Materials and methods

### Chemicals

Paraquat dichloride (≥ 98%) was purchased from Sigma of St. Louis, MO, USA. Rapamycin was obtained as a yellow powder from Alfa Aesar Company through Thermo Fisher Scientific. Dimethyl sulfoxide (DMSO) was purchased from Sigma-Aldrich, St. Louis, MO, USA. Rapamycin was administered at a dosage of 2.5 mg/kg/day, dissolved in 1% DMSO (at a volume of 2.5 ml/kg), and was given to mice once daily for 14 days. The daily dosage of rapamycin was chosen based on the most recent animal body weight.

### Animals

Eight-week-old female C57BL/6J mice, weighing between 25 and 35 g, were utilized in this study. The mice were housed in the Medical Research Center (MASRI) of the College of Medicine at Ain Shams University in Cairo, Egypt. The animals were kept in a dedicated stainless-steel cage at a temperature of 22 ± 2 °C, with a 12-hour light/12-hour dark cycle. They were given unrestricted access to water and given standardized chow whenever they wanted it. Approval number (R72/2025) from the Animal Care and Use Committee (ACUC) and the Research Ethical Committee of the Faculty of Medicine at Ain Shams University ensures that this study complies with all applicable ethical standards. This study was performed in compliance with the ARRIVE guidelines 2.0.

### Isolation and culture of mice adipose tissue-derived mesenchymal stem cells (AT-MSCs)

AT-MSCs were isolated at the Faculty of Medicine, Ain Shams University, in the Histology Department’s stem cell research unit. Eight male C57BL/6 mice, weighing between 12 and 16 g at 8 weeks of age, were used to extract adipose tissues and processed for isolation and culture of AT-MSCs following the standard protocol described by Atia and Alghriany [20]. To prevent the potential impact of menstrual hormones on MSC viability and secretory capacity in female mice, we utilized exclusively male animals in our study. Ketamine (100 mg/kg, Woerden, Netherlands) and xylazine (7 mg/kg, Alfazyne, Woerden, Netherlands) were given intraperitoneally (i.p.) to the animals for analgesia. Then, mice were sacrificed by cervical dislocation to confirm death before AT-MSC preparation. After euthanasia, meticulously removing the adipose tissues from the omentum and epididymis areas, they were chopped into pieces measuring 1–2 mm^3^. They were then digested in a solution containing 0.2% type II collagenase while being shaken at 37 °C for 40 min. Afterward, a 25-ml pipette was used to triturate the tissue clumps for 2–3 minutes, and then a 70 μm nylon net was used for filtering. In a humidified environment with 5% (v/v) CO2 at 37 °C, the cells were centrifuged at 157× g for 10 min and subsequently transferred to DMEM low glucose media supplemented with 100 mg/mL of streptomycin, 100 units/mL of penicillin, and 10% fetal bovine serum (FBS; Gibco, NY, USA). The flasks used for cell culture were T75 from Grieber Bio-One in Germany. It was called “passage 0” when the primary cell culture was first initiated. The cultures were incubated for 48 h, then rinsed with PBS and maintained in the stromal media. After 24 h, the non-adherent cells were removed from the medium, and a new medium was introduced. On a triweekly basis, the culture medium was replaced. Following two washes with phosphate buffer saline (PBS), after significant colonies (80 ~ 90% confluence) had formed, the cells were detached by incubating them with 0.25% trypsin in 1 mM EDTA for 5 min at 37ºC. The trypsin was acquired from Gibco, NY, USA. Standard methods of trypsinization were used to subculture them twice to achieve enough cells. After around 10 days, the cells had grown to confluence. In the second passage, cells were collected, and their numbers were determined with a hemacytometer. Following that, 2 × 10^6^ cells/ml of viable cells were subcultured [20]. Adhesiveness and fusiform shape were characteristics of AT-MSCs in culture. The stem cells used in this experiment were of the third passage. To characterize the adhering cells, we eliminated the suspended cells and examined them using phase contrast and transmission electron microscopy, immunostaining, and flow cytometry.

#### Transmission electron microscopy examination of AT-MSCs

On the ninth day after initial culture, the cultured AT-MSCs were examined ultrastructurally. The cultivated cells were fixed for 1 h with 2.5% glutaraldehyde before rinsing with PBS (pH 7.4). After being saturated with 1% osmium tetroxide, they underwent dehydration using progressively stronger alcohols. Following this, they were rinsed and then embedded in epoxy resin. A 60 nm thickness was used for the ultrathin sections, which were then stained for 10 min with 2% uranyl acetate and another 10 min with lead citrate [21]. At Ain Shams University’s Faculty of Science, images were taken using a JEM-1200 EX II Electron Microscope (Jeol, Tokyo, Japan).

#### Streptavidin-biotin immunoperoxidase technique for the characterization of AT-MSCs

Characterizing AT-MSCs using the Streptavidin-biotin immunoperoxidase technique was performed by Nawah Scientific Inc. (Mokattam, Cairo, Egypt). CD44 (Cat. No. 550538), CD105 (Cat. No. 550546), and CD45 (Cat. No. 550539) were purchased from BD Pharmingen, San Diego, CA, USA. After the AT-MSCs from passage 3 (P3) were fixed in the dishes using an acetone/methanol combination, 2 ml of 3% H2O2 was added and left to halt endogenous peroxidase for fifteen minutes. The tissue culture Petri dishes were immersed in a warmed citrate buffer solution (pH 6) to retrieve the antigens. The dishes were then heated in a microwave at two watts for 10–20 min, cooled at room temperature for 20 min, and finally rinsed with distilled water (DW). The 30-F11 antibody should be diluted from 1:10 to 1:50 before use in a three-step staining procedure with biotin-conjugated anti-rat IgG2b (Cat. No. 550327) as the secondary antibody, Streptavidin-horseradish peroxidase (Cat. No. 550946), and the diaminobenzidine (DAB) detection system (Cat. No. 550880) for optimal indirect immunohistochemical staining. A brown hue was achieved by ten minutes of development with one or two drops of DAB. Finally, use DW and a blotting motion to clean the dishes. The colour brown was observed in the positive cytoplasmic responses.

#### Flow cytometric analysis for the characterization of adipose tissue-derived mesenchymal stem cells (AT-MSCs)

Flow cytometry was performed by Nawah Scientific Inc. (Mokattam, Cairo, Egypt). To identify the stem cell properties of the possible AT-MSCs, FACS analysis was used to examine the expression of specific surface antigens [20]. Using flow cytometry, five AT-MSC cultures were examined at P3 for the stem cell surface markers CD44, CD34, CD90, CD73, and CD105. The cultures were derived from the omentum and epididymis fat depots. In brief, separate microtubes were used to suspend 4 × 10^4^ cells in Stain Buffer (BD Biosciences, USA) after trypsinization and centrifugation. A series of fluorescein isothiocyanate (FITC)-conjugated anti-mouse monoclonal antibodies from BD Pharmingen was added to the samples and left to incubate at 37 °C for 20 min in the dark. The antibodies included CD44-FITC (Cat. No. 561859), CD34-FITC (Cat. No. 553733), CD90-FITC (Cat. No. 561973), CD73-FITC (Cat. No. 567701), and CD105-PE (Cat. No. 550546). The cells were examined using a BD FACSuite flow cytometer (BD Biosciences, USA) after the incubation period, and the data were prepared using the software FLOWJO.

### Experimental design

**Donor group 8**; male mice were utilized to harvest AT-MSCs, as previously stated.

#### Experimental groups: four groups were created at random from the 50 female mice

**Group I (Control Group):** Twenty animals, corresponding to the experimental groups, were given the drug solvent via the same means and for the same lengths of time. The four sets of five mice each were then randomly divided into identical subgroups.

**Subgroup Ia** (**PQ vehicle)**: Mice treated with PQ vehicle received a single i.p. injection of 0.9% saline.

##### Subgroup Ib (AT-MSC vehicle)

Mice were injected once intravenously (i.v.) with 200 µL of PBS (the AT-MSC vehicle) through the tail veins, and after two weeks, they were sacrificed.

**Subgroup Ic (**Rapamycin **vehicle)**: Mice treated with rapamycin vehicle received 2.5 mg/kg daily of 1% DMSO orally once daily for two weeks before being sacrificed.

##### Subgroup id (AT-MSCs control)

Mice were administered an i.v. Injection of 200 µL of AT-MSCs (1.0 × 10^5^ cells/mouse) once, and after two weeks, they were killed.

**Group II (Lung fibrosis)**: Pulmonary fibrosis was induced by injecting 40 mg/kg body weight of PQ i.p. once. The dosage of PQ was based on preliminary experiments showing the induction of acute lung injury [3].

**Group III (PQ + AT-MSCs)**: one day after PQ injection, animals were administered an i.v. njection of 1.0 × 105 AT-MSCs cells/mouse in 200 µL PBS through the tail vein and sacrificed four weeks later [22]. Except for two mice that were sacrificed three days after receiving AT-MSCs injection to determine where the cells migrated in the lung tissue.

**Group IV (PQ+ Rapamycin):** Animals received the same treatment identically with group II, after that, 2.5 mg/kg/day of rapamycin was dissolved in 1% DMSO and administered by oral gavage for two weeks, starting one day after PQ injection [18].

### Sample collection and processing

The animals in the experimental groups and the corresponding control mice were euthanized via cervical dislocation to verify death under general i.p. anesthesia using sodium pentobarbital (100 mg/kg) [23]. After euthanasia, lung tissues were acquired using a ventral midline incision, exposure, dissection, and quick excision for biochemical, histological, immunohistochemical, and Y chromosome identification purposes. The bodies of the dead animals will be discarded in the incinerator. Following the Guidelines for Care and Use of Laboratory Animals and with approval from the Animal Ethical Committee of Ain Shams University, all animal procedures will be performed.

### Histological study

#### Light microscopic study

After fixing, clearing, and dehydrating the lower lobes of each animal’s right lung, they were embedded in paraffin wax. The general architecture of the lung was examined by cutting five µm-thick sections and staining them with hematoxylin and eosin (H& E). Collagen fibers were identified using Masson trichrome stain, reticular fibers were identified using Gordon and Sweet’s silver impregnation staining method, and P53, a marker of apoptosis, was stained immunohistochemically [24].

#### Immunohistochemical study

The 4–5 μm paraffin sections were deparaffinized using xylene, and the tissue was rehydrated using alcohol at increasingly higher concentrations. Sections were subjected to a 10-minute boiling in a 10-millimolar citrate buffer (AP9003) with a pH of 6 to remove antigens. Then, they were incubated with the primary antibody for an hour. This was done to prepare them for immunohistochemical staining. After immersing the sections in 0.1% H_2_O_2_ for 15 min, the endogenous peroxidase enzyme was blocked. To prevent a strong background, the sections were washed with phosphate buffer solution and then incubated in antiserum for 5 min at room temperature. The primary antibody, a goat polyclonal antibody to P53 (MBS448112) from MyBioSource, Inc. in San Diego, USA, was supplied at a dilution of 1/200 and is an apoptotic marker. The immunostaining process was carried out using an Ultravision detection system (TP-015-HD), while Mayer’s hematoxylin (TA060-MH) was employed for the counterstaining. Based on the data of the antibody manufacturer, the reactions appeared cytoplasmic for P53. Negative controls were established by omitting the addition of the primary antibody.

#### Transmission electron microscopic study

After fixing the left lung sections in 0.1 ml of cacodylate buffer at pH 7.4 for 24 h at 4 °C, the tissue was rinsed with distilled water containing 1% osmium tetroxide. The parts were immersed in epoxy resin after dehydration. Thin slices of 1 μm were cut and stained with toluidine blue. For analysis, ultrathin slices (80 nm) were mounted on grids and stained. The specimens were examined and photographed using a JEM-1200 EX II Electron Microscope (Jeol, Tokyo, Japan) [25]. Earlier steps, including section preparation (both semithin and ultrathin) and section analysis (staining), were carried out at the Unit of Electron Microscopy, Ain Shams University’s Faculty of Science.

### Histomorphometric study

Ten non-overlapping randomly selected fields (x40) from different areas of each rat in each group were analyzed to quantify the area percentage of collagen, the area percentage of reticular fibers, and the area percentage of P53-positive cells. Measurements were conducted utilizing the “Leica Qwin 500 C” image analysis computer system, Ltd. (Cambridge, England) at the Histology Department, Faculty of Medicine, Ain Shams University.

### Tissue homogenization

The left lower lung samples were disrupted and homogenized using a rotor-stator homogenizer called the Tissue Ruptor II (Qiagen, Hilden, Germany). In 15–90 s, depending on the sample size and toughness, it can simultaneously homogenize and disrupt a single tissue sample in a lysis buffer. After that, the mixture is spun at 4000 rpm for 20 min. Lastly, biochemical evaluation and RNA extraction are performed on the cell supernatant.

### Biochemical assessment

Mice were tested for oxidant-antioxidant status by assessing malondialdehyde (MDA) levels, superoxide dismutase (SOD) activity, and reduced glutathione (GSH) in lung tissue. This was done by extracting, homogenizing, and centrifuging tissues from the left lower lobes of the lungs. The activity of MDA was measured using the Yeginsu and Ergin method [26]. The results were given in nanomoles per gram of protein. The method of Lu and Finkel was used to measure SOD activity [27]. Its findings were expressed in units per gram of protein. The results were represented as milligrams per gram of protein and were obtained by measuring reduced glutathione (GSH) using the Jurczuk et al. method [28]. We measured the levels of inflammatory cytokines in the lung tissues of the mice by using enzyme-linked immunosorbent assay (ELISA) kits from the Nanjing Jiancheng Bioengineering Institute in China, following the manufacturer’s instructions. These cytokines include interleukin-6 (IL-6) and tumor necrosis factor-alpha (TNF-α). Using ELISA kits, we were able to determine the amounts of IL-6 and TNF-α in the supernatants. All laboratory analyses were carried out at the Department of Clinical Pathology & Immunology, Ain Shams University Hospital.

### Detection of the Y chromosome (SRY gene) in the mouse lung tissue

Gene amplification analysis of the Y chromosome and agarose gel electrophoresis were employed to identify male AT-MSCs from lung tissues. The expression level of the Y chromosomal gene was amplified from mRNA using a sex-determining region Y-specific primer to target the sex-determining region Y gene, NCBI reference sequence: NM_001416740.1, utilizing the QuantiTect SYBR Green PCR Kit, catalog number: 204141 (Qiagen, Germany). SRY DNA detection was conducted with the subsequent primers: forward primer Sequence (5’ TTTATGGTGTGGTCCCGTGG3’) and reverse (3’ GTTGAGGCAACTTCACGCTG 5’), with a product size appeared at 254 base pairs. Tissue homogenate with ethanol was placed onto the RNeasy Mini (cat no: 74104, *Qiagen*,* Hilden*,* Germany*) spin column [29]. The manufacturer’s protocol was followed for RNA extraction and purification. The QuantiTect Reverse Transcription Kit (205310, Qiagen, Hilden, Germany) reverse transcription. After cooling the reverse-transcription reactions, real-time PCR was performed. QuantiTect primer assay [Rn_Arxes2, QuantiTect Primer Assay, Gene Globe ID: QT02442419 (cat no: 249900, Qiagen, Germany)] and QuantiTect SYBR Green PCR Kit cat no: 204,141 amplified Y chromosomal gene expression from mRNA. To activate HotStarTaq DNA Polymerase, the real-time cycler activation step was set to 15 min at 95ºC. Three-step cycling: 15 s denaturation at 94ºC, 30 s annealing at 55ºC, and 30 s extension at 70ºC for 40 cycles. Furthermore, expression levels were normalized to β-actin levels, serving as a reference gene. The relative expression level (fold change) of the Y chromosome was normalized to β-actin and compared to a negative control sample using the 2-∆∆Ct equation [30].

For agarose gel electrophoresis, a 1% agarose gel is prepared by dissolving agarose 1-gram (*Sigma Aldrich*,* USA*) powder in a 100 mL Tris-borate-EDTA (TBE) buffer, heating the mixture until the agarose is completely melted, and then staining with Ethidium bromide. The solution is then cooled slightly and poured into a gel casting tray with a comb to create wells. Once the gel has solidified, the DNA samples, mixed with a 6X DNA loading dye, are loaded into the wells. The gel is placed in an electrophoresis chamber filled with the same buffer used to prepare the gel. An electric voltage was adjusted to 95 mV, causing the negatively charged DNA molecules to migrate towards the positive electrode, resulting in separation by size. After electrophoresis, the DNA is visualized under UV light by UVP Transilluminator (Analytic Jena, Endress& Hauser, Germany). The 1 Kbp DNA Ladder RTU, range 250–10,000 base pairs, cat no: DM010-R500 (GeneDirex, UK) was used as a marker for DNA bands.

### Statistical analysis

The data was analyzed statistically using one-way analysis of variance (ANOVA) and post-hoc least significant difference (LSD) testing. The statistical package used was SPSS for Windows, version 26, developed by IBM Inc. of Chicago, Illinois, USA. The values were defined as statistically significant as p-value < 0.05. The summary of the data was expressed as mean ± standard deviation (SD). All statistical graphs were performed using GraphPad Prism 10.0.

## Results

### AT-MSCs expansion and characterization results

**Fig. 1:**
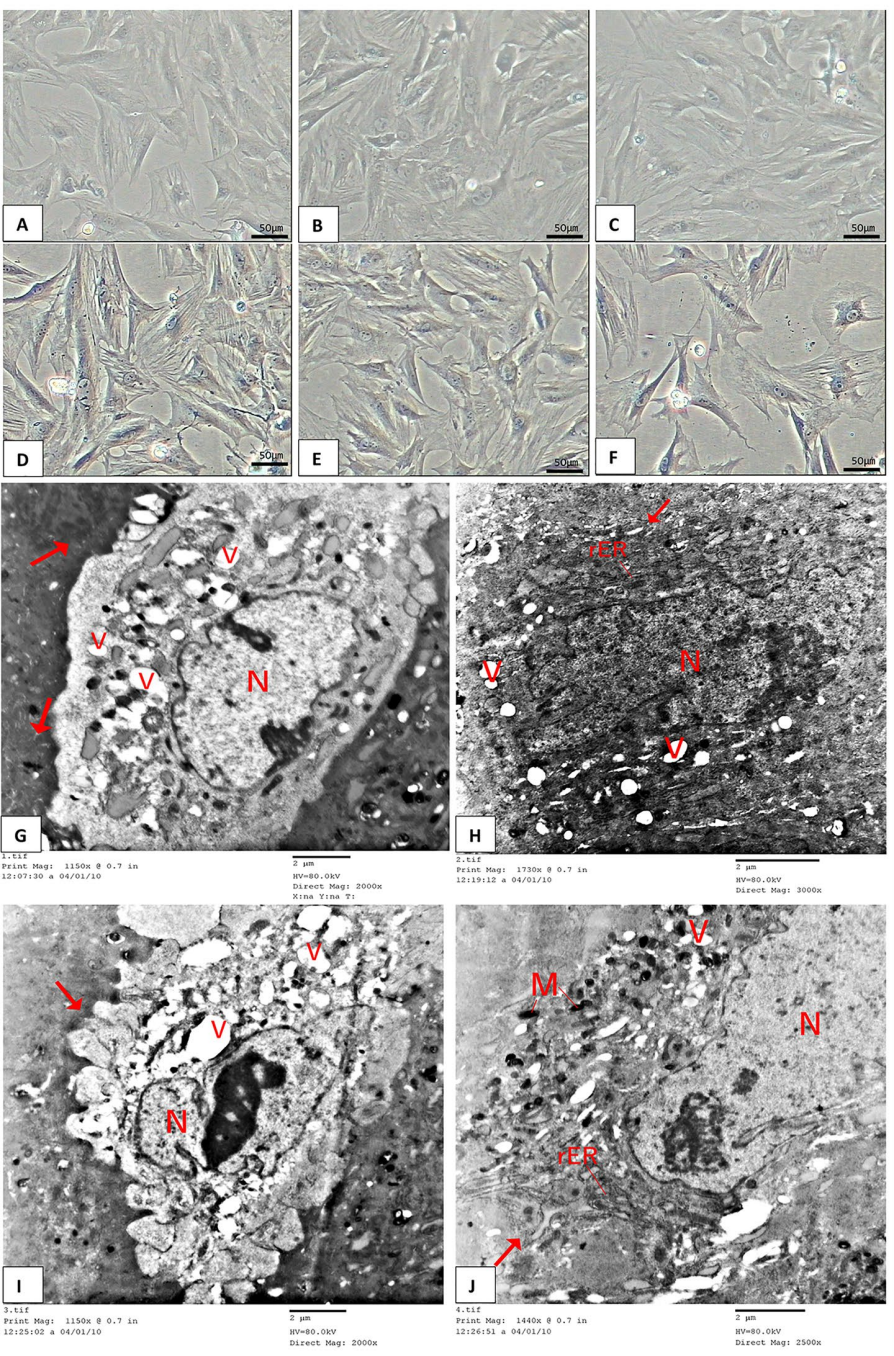
Morphology of adipose-derived mesenchymal stromal cells (AT-MSCs). (**A**-**F**) **A** photomicrograph of mice AT-MSCs *via* phase-contrast microscopy. AT-MSCs culture from adipose tissue exhibiting fibroblastic morphology in the third passage. Colonies of attached cells with vesicular nuclei and interdigitating processes are noticed. (Phase contrast, A-D x20). (**G**-**J**) Transmission electron microscope images of AT-MSCs at day 10 (P3). (**N)**: Nucleus, (red arrow): blebs, (V): intracellular vesicles, (**M**): mitochondria, (rER): rough endoplasmic reticulum (TEM, Scale bar = 2 μm)

AT-MSCs were non-adherent and rounded with varied morphologies on day one of primary culture. On culture day three, adhering cells extended short cytoplasmic processes. Other cells refracted and didn’t adhere. Round cell colonies appeared occasionally. Fibroblast-like cells appeared on day seven of cultivation. They resembled long interdigitating processes. They have vesicular nuclei and granular cytoplasm. Some have multiple nucleoli (Fig. 1A-F).

TEM examination of the AT-MSC surface exhibited filopodia or blebs, accompanied by dense bodies and cytoplasm filled with rough endoplasmic reticulum, Golgi apparatus, free ribosomes, mitochondria, and intracellular vesicles. Nuclei possess euchromatin, typically featuring a prominent nucleolus, indicative of an active protein-synthesizing cell; in certain cells, the nucleus exhibited convolutions (Fig. 1G-J).

**Fig. 2:**
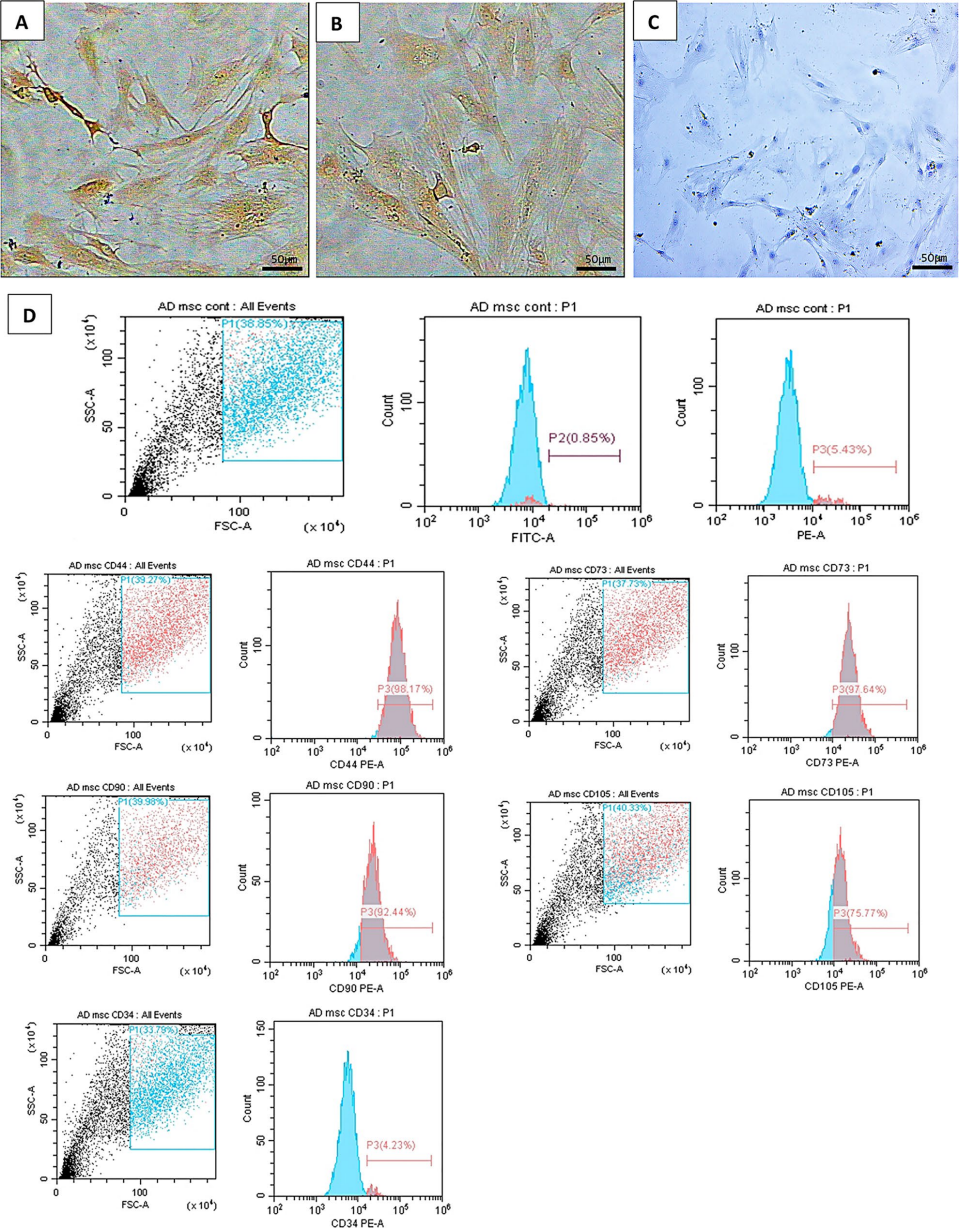
Immunohistochemical reaction of cell-surface antigens of mice AT-MSCs showed (**A**) a positive reaction for CD44, (**B**) a positive reaction for CD105, and (**C**) a negative reaction for CD45 (Phase contrast, A-C x20). (**D**) Flowcytometric analysis of cell-surface antigens of mice AT-MSCs showed a Gate in the cell population, blue line– Controls (isotype or secondary antibody); Red line– Cell surface markers. A positive reaction for CD44, CD73, CD90, and CD105 (mesenchymal cell markers) and a negative reaction for CD34 (hematopoietic cell marker). FITC: Isotype-Fluorescein isothiocyanate, PE: Isotype-Phycoerythrin

In the streptavidin-biotin immunoperoxidase assay, the majority of the P3 subculture cells showed positive brownish cytoplasmic reactions for CD44 and CD105 (Fig. 2A and B, respectively), whereas no cells showed any positive reactions for CD45(Fig. 2C). Furthermore, AT-MSC cell surface markers were examined using flow cytometry. Figure 2D shows that the isolated AT-MSCs were very pure, as evidenced by the positive expression of CD44 (98.14%), CD73 (97.64%), CD90 (92.44%), and CD105 (75.77%). In contrast, CD34 showed only a negative expression of 4.23%.

### Histological results

#### Morphological evaluation of the lung tissue

##### Hematoxylin and Eosin-stained sections

**Fig. 3:**
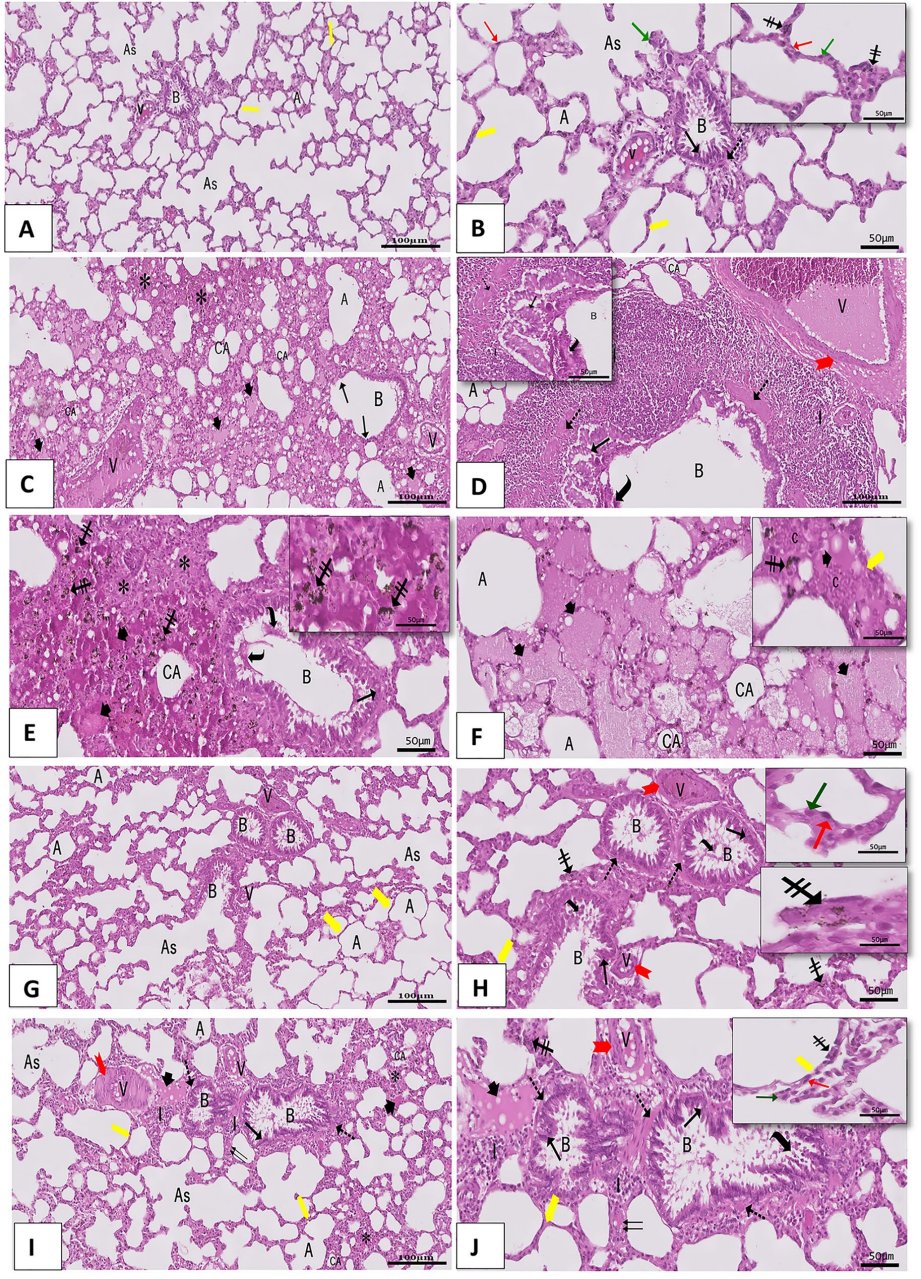
A photomicrograph of a hematoxylin and eosin of a lung section showing: (**A**&**B**) The control group shows normal bronchiole (B), many alveoli (A)separated by thin interalveolar septa (thick yellow arrow), and adjacent blood vessels (V). An alveolar sac is also seen (As) as several connected pouches.Bronchiole (B) is structured in a typical folded simple columnar ciliated epithelium (black arrow), encircled by smooth muscle fibers (dotted arrow). Thealveoli (A) show pneumocyte type I (red arrow), type II (green arrow), and a few dark brown particles (crossed arrow) in the thin interalveolar septum(thick yellow arrow). (H&E, Ax10, Bx20, inset x40). (**C**-**F**) Paraquat (PQ) group (Pulmonary fibrosis group) shows (C) interstitial lung consolidation (*), distortedvacuolated epithelial cells of the bronchiolar mucosa (black arrow), and heavy inflammatory cell infiltration (I) are detected. Most of the alveoliare collapsed (CA), while others are dilated (A). (**D**-**F**) Discontinuity of both bronchiolar mucosa (black arrow) and bronchiolar muscular coat (dot arrow)is noticed. The bronchiolar lumen contains sloughed bronchiolar mucosal cells with dark, small nuclei (curved arrow), bathed in eosinophilic exudate(arrowhead). Heavy peribronchiolar and perivascular inflammatory cellular infiltration (I) is also noticed. Notice the congested blood vessels (V) as well asthe blood vessels with a thickened muscular wall (red bifid arrow). (**E**& **F**) Acidophilic homogenous material (arrowhead) is detected in the lung interstitium.Dark brown particles (crossed arrow) are seen deposited in the interstitium and the alveolar cavities. Alveolar cavities are also occupied by extravasatedRBCs (C). The interalveolar septum (thick yellow arrow) appears thickened, containing congested dilated capillaries (C) with Dark brown particles(crossed arrow). (H&E, C&Dx10, E&Fx20, inset x40). (G &H) The AT-MSCs-treated group reclaims almost all its normal histological features. Intact continuousbronchiolar (B) layers are noticed, which are formed of bronchiolar mucosa (black arrow) and bronchiolar muscular coat (dot arrow) with few sloughedbronchiolar mucosal cells (curved arrow). Notice the patent alveoli (A) that are surrounded by type I (red arrow) and type II pneumocytes (green arrow).However, a few congested blood vessels (V) are still present with thin walls (red bifid arrow) within thin Interalveolar septa (thick yellow arrow). Notice thata few dark brown particles (crossed arrow) are seen deposited in the interstitium. (H&E, Gx10, Hx20, inset x40). (**I** &**J**) The Rapamycin-treated -treated groupreveals areas of patent alveoli (A) side by side with collapsed ones (CA) and consolidated parts (*). Interalveolar septa appeared with variable thickness;some appear thin (thick yellow arrow), and some appear thick (↑↑). Intact bronchiolar (B) mucosa (black arrow) with a continuous muscular layer (dotarrow) and few sloughed bronchiolar mucosal cells with dark small nuclei are noticed. Stand-still congested blood vessels (V) with thickened walls (bifidred arrow) and inflammatory cellular infiltrations (I) are noticed. Few acidophilic homogenous materials (arrowhead) are detected in the lung interstitium.A few dark brown particles (crossed arrow) are seen deposited in the interstitium. The alveoli show pneumocyte type I (red arrow), type II (green arrow),and a few dark brown particles (crossed arrow) in the thin interalveolar septum (thick yellow arrow). (H&E, Ix10, Jx20, inset x40)

The control group’s normal lung parenchyma was shown by H&E-stained lung sections, which showed that the bronchioles, alveolar sacs, alveoli, and interalveolar septa were structurally intact. Normal bronchiolar folded simple columnar ciliated epithelium, with the surrounding thin, smooth muscle fibers coat, was observed. The alveoli showed numerous flat cells (pneumocytes type I), rounded cells protruding into the alveolar lumen (pneumocyte type II), and few dark brown particles in the thin interalveolar septum **(**Fig. 3A&B).

Regarding the Paraquat (PQ) group (Pulmonary fibrosis group), there was severe destruction to the lung architecture. On examination, consolidation of the lung interstitium, distorted vacuolated epithelial cells of the bronchiolar mucosa, and heavy inflammatory cellular infiltration were detected. Collapse of some alveoli was observed, while emphysematous dilatation of others was noticed. Discontinuity of both bronchiolar mucosa and bronchiolar muscular coat was noticed. The bronchiolar lumen contained sloughed bronchiolar mucosal cells with dark, small nuclei bathed in eosinophilic exudate. Heavy peribronchiolar and perivascular inflammatory cellular infiltration was also noticed. Congested blood vessels with thickened muscular walls were observed. Acidophilic homogenous material was detected in the lung. Massive dark brown particles were seen deposited in the interstitium. The interalveolar septum appeared thickened and occupied by extravasated RBCs (Fig. 3C-F).

By using the AT-MSCs in group (III), lung tissue reclaimed almost all its normal histological features. Intact continuous bronchiolar layers were noticed, which were formed of intact bronchiolar mucosa and continuous bronchiolar muscular coat with few sloughed bronchiolar mucosal cells. Patent alveoli, bordered by pneumocytes type I and pneumocytes type II, were noticed. However, a few congested blood capillaries were still present with thin walls within thin Interalveolar septa. Notice that a few dark brown particles were seen deposited in the interstitium (Fig. 3G&H).

While the Rapamycin-treated group revealed areas of patent alveoli side by side with collapsed ones and consolidated parts, interalveolar septa appeared with variable thickness; some appeared thin, and some appeared thick. Intact bronchiolar mucosa with a continuous muscular layer and few sloughed bronchiolar mucosal cells with dark, small nuclei were noticed. Stand-still congested blood vessels with thickened walls and inflammatory cellular infiltrations were noticed. Few acidophilic homogenous materials were detected in the lung interstitium. A few dark brown particles were seen deposited in the interstitium. The alveoli showed pneumocyte type I and type II, and a few dark brown particles in the thin interalveolar septum (Fig. 3I&J).

### Masson’s trichrome-stained sections

**Fig. 4:**
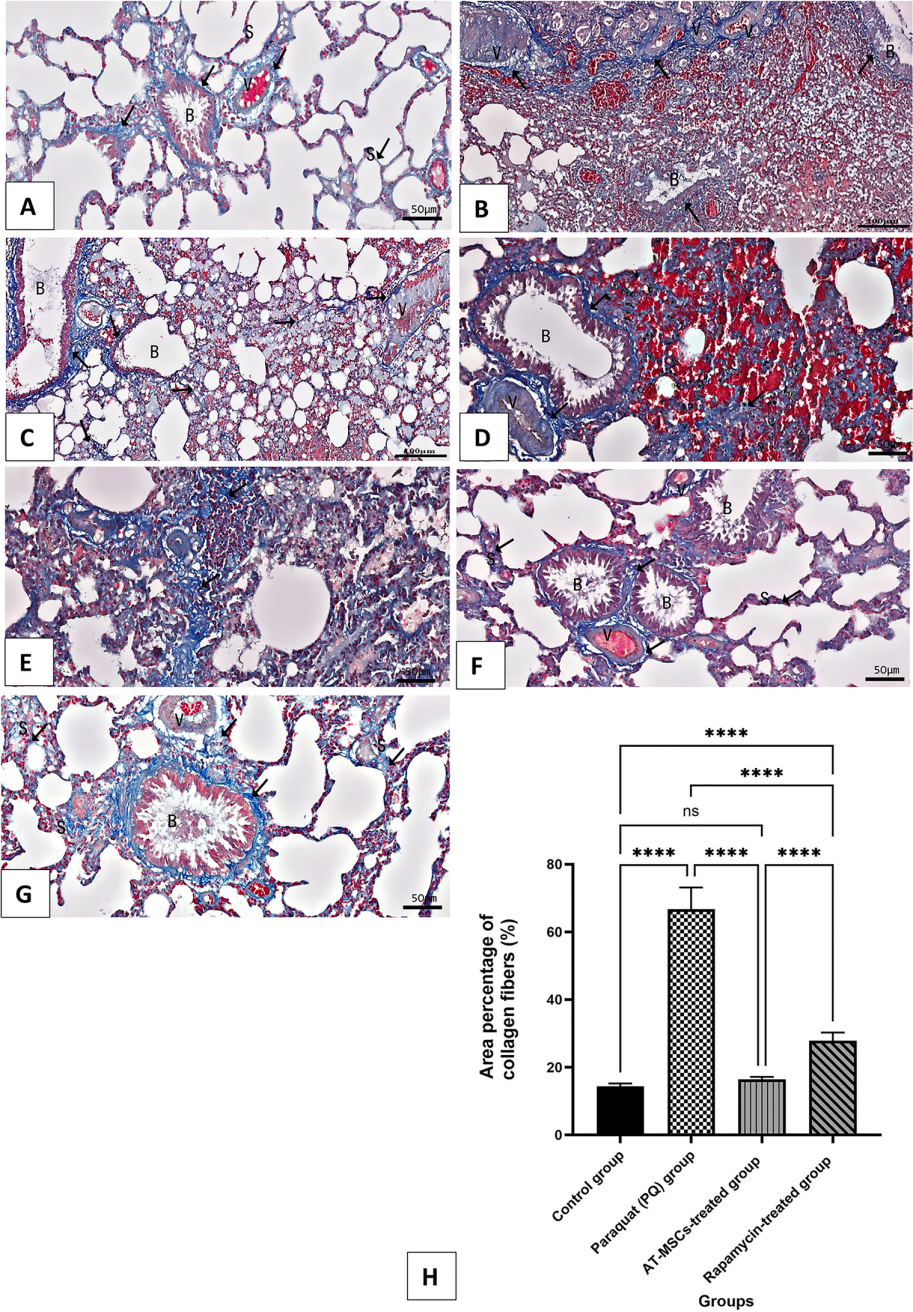
Masson’s trichrome-stained collagen fibers (↑) in: (**A**) the control group reveals fine collagen. (**B**-**E**) Paraquat (PQ) group (Pulmonary fibrosis group) shows massive deposition of collagen fibers. (**F**) The AT-MSCs-treated group reveals scattered fine collagen fibers. (**G)** The Rapamycin-treated group reveals a moderate amount of collagen deposition. (S) interalveolar septa, (B) bronchioles, and blood vessels (V). (Masson’s trichrome, A, D-Gx20, B&Cx10). (**H**) The statistical results are shown as mean ± standard deviation (SD). ns: non-significant and **** significant at *P* < 0.0001

The data of the experimental groups’ Masson’s trichrome-stained sections were displayed in Fig. 4; Table 1. In the control group, fine collagen fibers were seen in the interalveolar septa, bronchiole adventitia, and blood vessel adventitia (Fig. 4A). In the pulmonary fibrosis model group II, obvious deposition of collagen fibers in the thickened interalveolar septa as well as in the adventitia of bronchioles and blood vessels was noticed (Figs. 4B-E). Meanwhile, sections of AT-MSCs treated group III exhibited dispersed fine collagen fibers (Fig. 4F). Rapamycin-treated group IV had a moderate level of collagen deposition (Fig. 4G). Group II (Pulmonary fibrosis) treated with Paraquat (PQ) and group IV (rapamycin) showed a marked increase in the percentage of collagen deposition, especially around blood vessels and bronchioles. There was a statistically significant reduction in deposition of collagen in group III treated with AT-MSCs compared to the PQ group, but no difference compared to the control group. Rapamycin-treated group IV showed significantly lower collagen deposition compared to the PQ group (Fig. 4H).

**Table 1:** Morphometric and biochemical results in all experimental groups. Data are expressed as mean ± std. Deviation (SD)

Mean ± Std. Deviation	Control group	Paraquat (PQ) group	AT-MSCs-treated group	Rapamycin-treated group	*P* Value
Number of mice per group	10	10	10	10	
Area percentage of collagen fibers (%)	14.42 ± 0.7772	66.69 ± 6.461	16.42 ± 0.7207	27.89 ± 2.398	< 0.0001 A-B 0.5796 A-C < 0.0001 A-D < 0.0001B-C < 0.0001B-D < 0.0001 C-D
Area percentage of reticular fibers (%)	18.60 ± 1.101	66.38 ± 3.524	20.90 ± 1.345	26.64 ± 4.258	< 0.0001 A-B 0.3001 A-C < 0.0001 A-D < 0.0001B-C < 0.0001B-D 0.0005 C-D
P53 lung	12.9 ± 2.601	68.2 ± 8.039	14.8 ± 1.398	33.8 ± 0.7888	< 0.0001 A-B 0.7571 A-C < 0.0001 A-D < 0.0001B-C < 0.0001B-D < 0.0001 C-D
Malondialdehyde (MDA, nmol/mg)	1.99 ± 0.3864	5.22 ± 0.6596	2.082 ± 0.3923	3.472 ± 0.3385	< 0.0001 A-B 0.9701 A-C < 0.0001 A-D < 0.0001B-C < 0.0001B-D < 0.0001 C-D
Reduced glutathione (GSH, ng/ml)	22.43 ± 3.186	11.67 ± 0.5165	20.7 ± 1.216	15.34 ± 1.496	< 0.0001 A-B 0.1884 A-C < 0.0001 A-D < 0.0001B-C 0.0006B-D < 0.0001 C-D
Superoxide dismutase (SOD, u/mg)	190.4 ± 3.471	115 ± 1.58	173.8 ± 5.007	152.5 ± 2.66	< 0.0001 A-B < 0.0001 A-C < 0.0001 A-D < 0.0001B-C < 0.0001B-D < 0.0001 C-D
Tumor necrosis factor–α (TNF-α, pg/mg tissue)	13.86 ± 0.5985	26.14 ± 0.8154	13.32 ± 0.7436	18.44 ± 0.8195	< 0.0001 A-B 0.3855 A-C < 0.0001 A-D < 0.0001B-C < 0.0001B-D < 0.0001 C-D
Interleukin -6 (IL-6, pg/mg tissue)	7.26 ± 1.158	15.44 ± 1.445	7.3 ± 1.198	10.95 ± 1.45	< 0.0001 A-B 0.9999 A-C < 0.0001 A-D < 0.0001B-C < 0.0001B-D < 0.0001 C-D

A: Control group, B: Paraquat (PQ) group, C: AT-MSCs-treated group, D: Rapamycin-treated group

### Gordon and Sweet’s silver impregnation-stained sections

Gordon and Sweet’s silver impregnation revealed the demonstration of reticulin fibers in a section of the lung tissue of the experimental groups. The reticular fibers in the lung tissue of the control group (GI) appeared delicate and filigreed, as is typical for a normal reticular network. Tiny, reticular fibers stained black are also found in the interstitial tissue and the walls of the alveoli, bronchioles, and blood vessels.

**Fig. 5:**
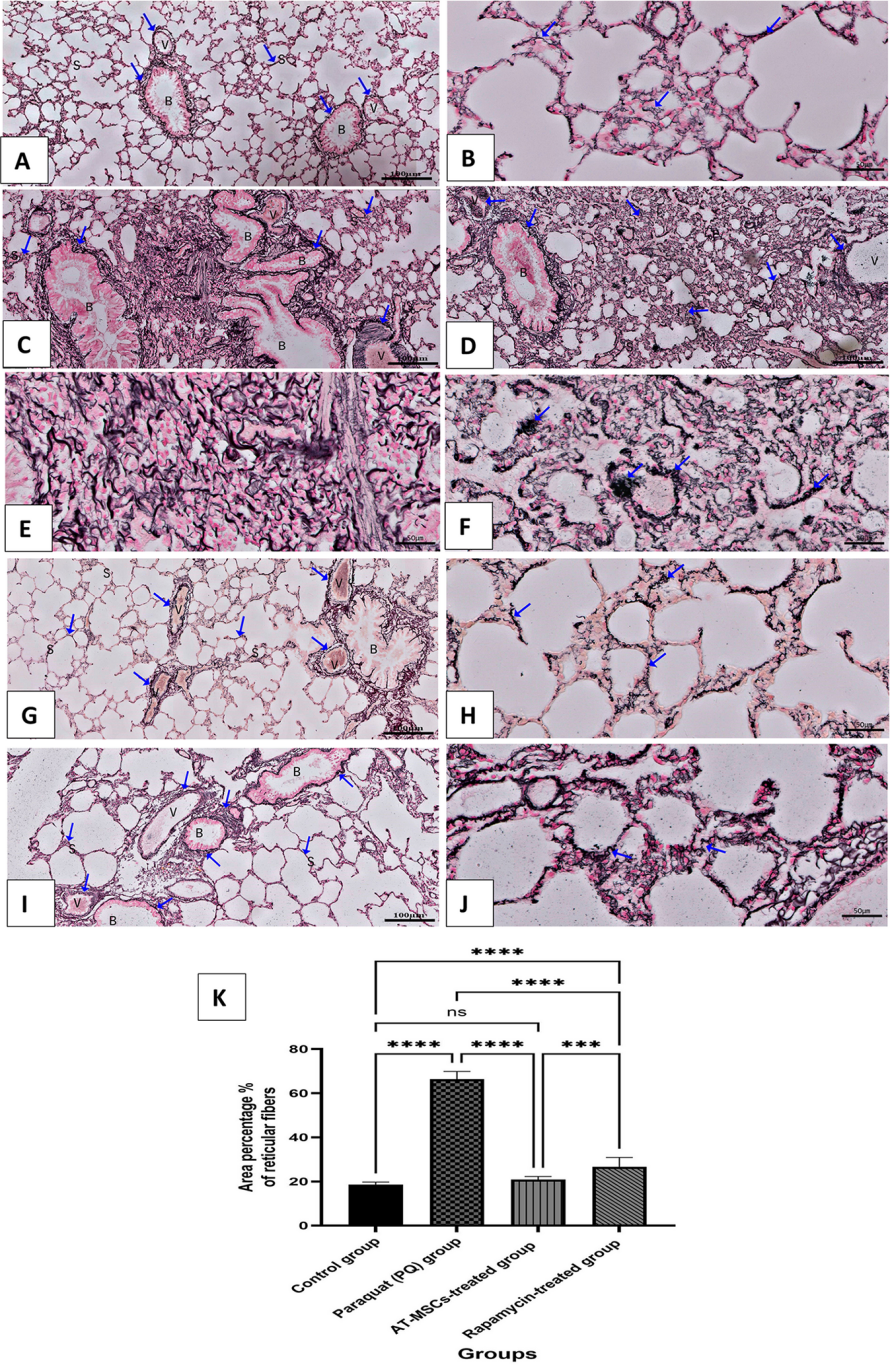
A photomicrograph of Gordon & Sweet’s silver-stained sections of (**A**&**B**) control group reveals that the reticular fibers (↑) appear fine, filigreed, and located in the network. In addition, fine black stained reticular fibres (↑) support the walls of the blood vessel (v), the interalveolar septum (S), the bronchioles (**B**), and in the interstitial tissue. (**C**-**F**) Paraquat (PQ) group (Pulmonary fibrosis group) shows massive deposition of the reticular fibers (↑) in the walls of the blood vessel (v), the interalveolar septum (S), the bronchioles (**B**), and in the interstitial tissue. Notice a well-developed irregular reticular fiber (↑) network in the areas with inflammatory infiltrate (I). (**G**&**H**) AT-MSCs treated group reveals scattered fine deposition of the reticular fibers (↑) in the walls of the blood vessel (v), the interalveolar septum (S), and that of the blood vessels (V). (**I**&**J**) The Rapamycin-treated group reveals a moderate amount of reticular fibers (↑) deposition in peribronchiolar (**B**), perivascular (V), and interalveolar septa (S). (Gordon & Sweet’s silver staining, **A, C, D, G**, Ix10, **B, E, F, H**, Jx40). (**K**) The statistical results are shown as mean ± standard deviation (SD). ns: non-significant, *** significant at *P* < 0.001, and **** significant at *P* < 0.0001

In the pulmonary fibrosis group (GII) of PQ mice, there is a buildup of reticular fibers in the interalveolar septa, which causes these areas to thicken significantly. Additionally, fibrosis progresses, and architectural abnormalities in the lung parenchyma are observed. Thick bundles with network disarray were discovered in all areas afflicted by fibrosis, and condensation of reticular fibers was observed in most lung sections due to their enormous accumulation. In areas where inflammation was present, a reticular fiber network was visible, which was developed but not perfectly regular. This is where the structure resembles lymphoid tissue rather than typical lung parenchyma. Group III treated with AT-MSCs, on the other hand, had fewer reticulin fibers in their lung tissue. Nevertheless, reticulin fibers in group IV treated with rapamycin showed a strong black staining affinity, which seemed to be higher than in groups GIII (AT-MSCs treated) and control (GI) (Fig. 5A-F). A comparison of the effects of rapamycin and AT-MSC therapy on the percentage of reticular fiber area in lung tissue stained with Gordon and Sweet’s silver impregnation stain across all experimental groups is shown in Table 1; Fig. 5G.

### Evaluation of immunohistochemical results of apoptosis marker (P53) expression in lung tissue

**Fig. 6:**
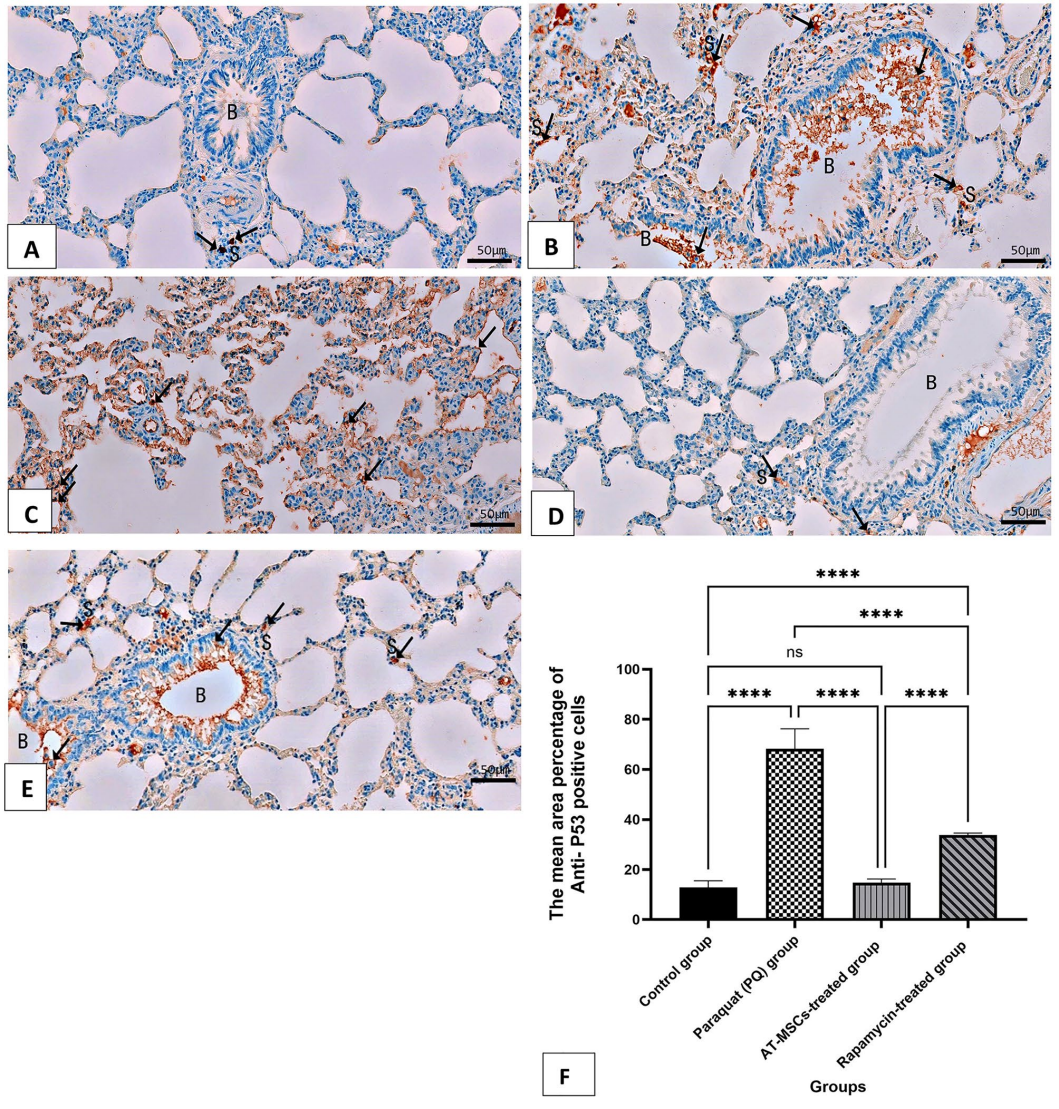
A photomicrograph of Immunohistochemical (IHC) stained sections of (**A**) the control group reveals faint + ve brown (↑) cytoplasmic reaction for P53 in the lung interstitium (S). (**B**,**C**) Paraquat (PQ) group (Pulmonary fibrosis group) shows strong + ve brown (↑) cytoplasmic immunoreaction for P53 on both sloughed bronchiolar (B) mucosal cells in the lumen of the bronchioles and in the alveolar wall (S). (**D**) The AT-MSCs-treated group revealed very few cells with + ve reaction for P53 in the form of brown cytoplasmic deposits (↑) in the alveolar walls (S). (**E**) The Rapamycin-treated group revealed a moderate cytoplasmic reaction (↑) for P53 in bronchiolar cells. Also, moderate + ve reaction in the alveolar walls (S) is noticed. (P53, A-Ex20). (**F**) The statistical results are shown as mean ± standard deviation (SD). ns: non-significant and **** significant at *P* < 0.0001

Data from P53 immunohistochemistry were displayed in Table 1; Fig. 6 for each of the experimental groups. The control group’s lung sections revealed a faint + ve cytoplasmic reaction for P53 in the lung interstitium (Fig. 6A). Paraquat (PQ) group (Pulmonary fibrosis group) showed massive strong + ve brown cytoplasmic immunoreaction for P53 on both sloughed bronchiolar mucosal cells in the lumen of the bronchioles and in the alveolar wall (Fig. 6B-C). The AT-MSCs-treated group revealed very few cells with + ve reaction for P53 in the form of brown cytoplasmic deposits in the alveolar walls (Fig. 6D). Rapamycin-treated group revealed a moderate cytoplasmic reaction for P53 in bronchiolar cells and in the alveolar walls (Fig. 6E). The mean area percent of P53 was significantly higher in groups II and IV compared to group I. Moreover, there was a significant reduction in group III vs. group IV. A non-significant increase was observed in group III vs. group I (Fig. 6F).

### Transmission electron microscopic histological results of the lung tissue

**Fig. 7:**
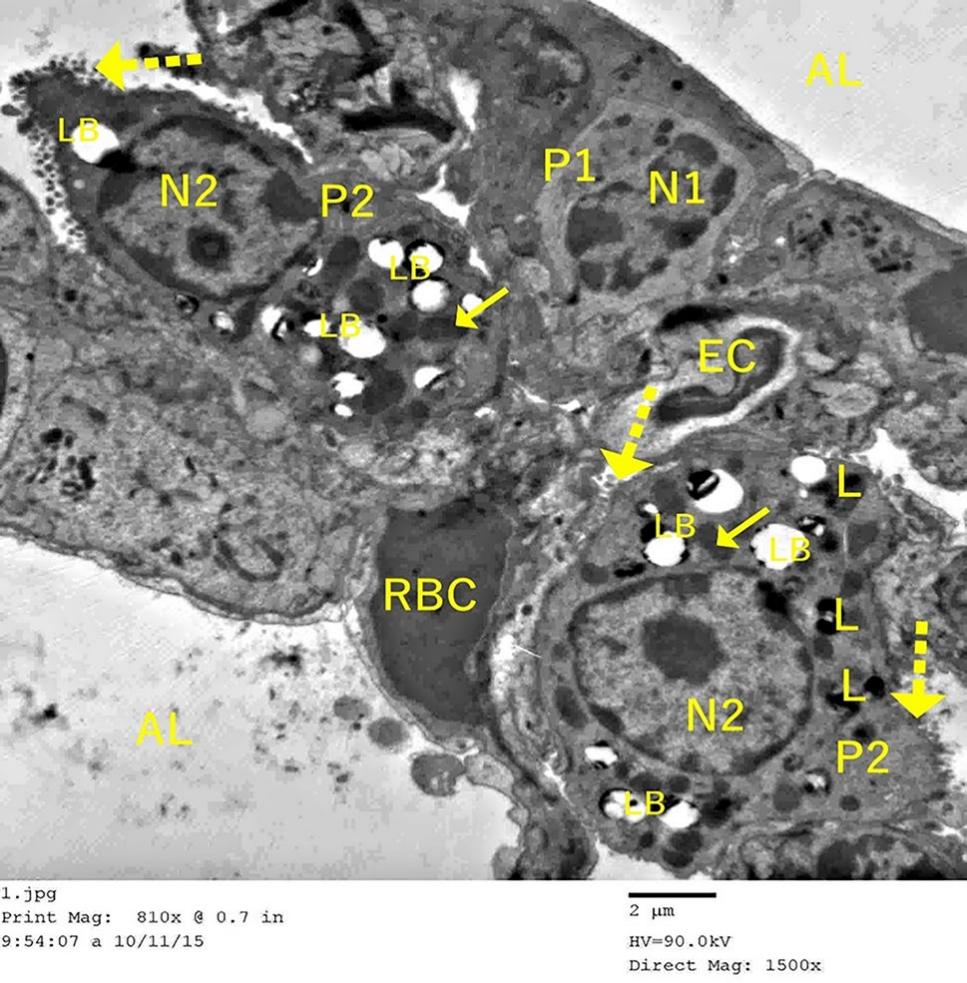
Electron micrographs of the ultrathin sections of control murine lung tissue demonstrate that Type I pneumocyte (P1) exhibits a regular contour and a euchromatic nucleus (N1). Patent alveolar space (AL) is noted. Red blood cells (RBC) are within the capillary lumen. Type II pneumocyte (P2) exhibits a microvillous border (dot arrow), mitochondria (↑), and a euchromatic nucleus (N2) oriented towards an open alveolar space. Type II cells display lamellar bodies (LB) and lysosomes (L). Pneumocyte type I, with its basal lamina joined to the endothelial cells (EC) of the adjacent blood capillary, is observed (TEM, Scale bar = 2 μm)

Mice in the control group (group I) showed classic alveolar architecture in ultrathin lung tissue sections, with the two types of pneumocytes surrounding the alveoli. The interalveolar septa showed blood capillaries. The basal lamina of a Type I pneumocyte was connected to the endothelial cells of the blood capillary, and the nucleus was elongated and surrounded by a thin layer of cytoplasm. Red blood cells were observed within the capillary lumen. A type II pneumocyte was characterized by a microvillous border and a spherical euchromatic nucleus that faced a patent alveolar space. Its cytoplasm contained lamellar bodies with concentric lamellae, lysosomes, and mitochondria (Fig. 7).

**Fig. 8:**
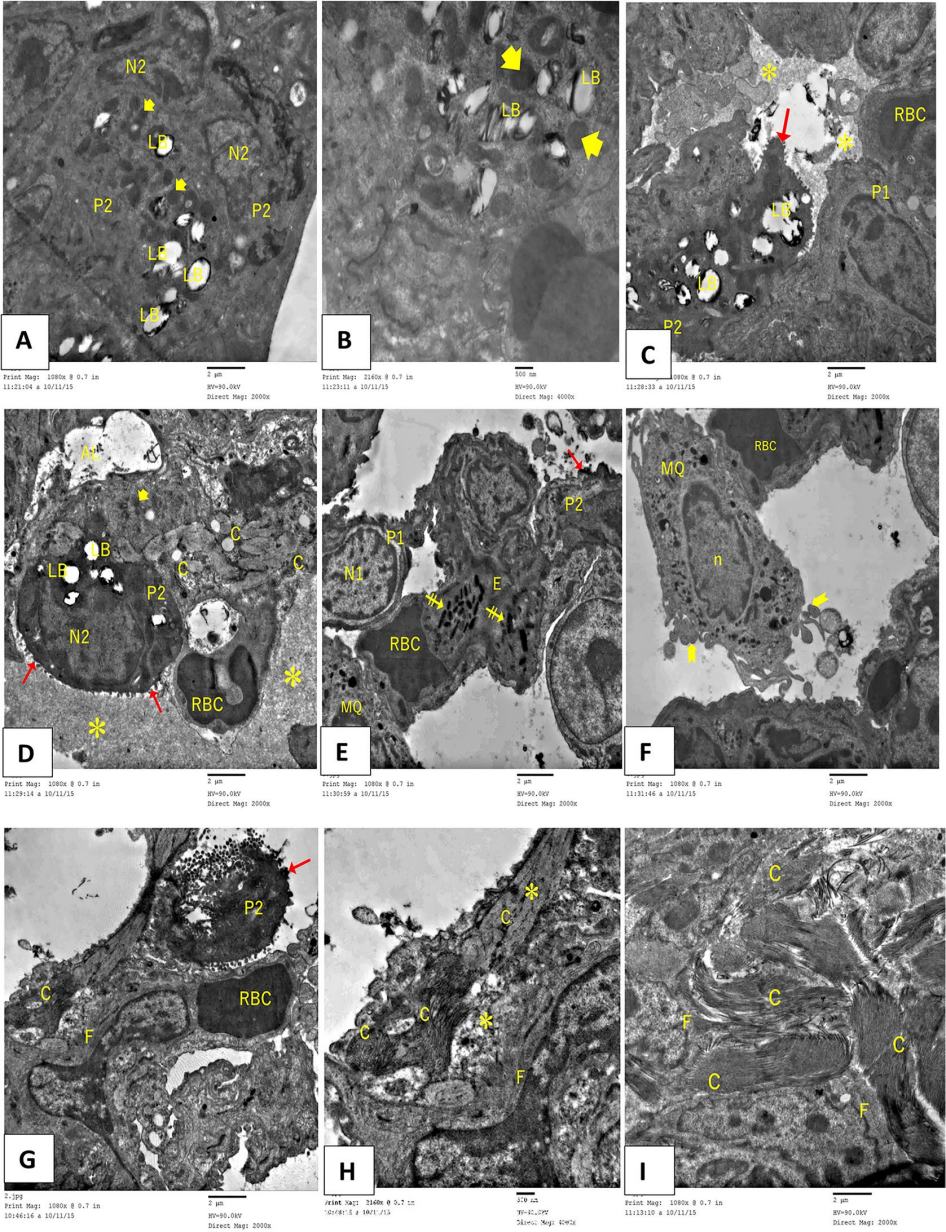
Electron micrographs of an ultrathin segment of Paraquat (PQ) (Pulmonary fibrosis) murine lung tissue revealing: (**A-F**) severe degenerative alterations in type I (P1) and type II (P2) pneumocytes are seen. Type II pneumocytes (P2) exhibit an irregular heterochromatic nucleus (N2), characterized by the absence of the typical lamellar structure, resulting in vacuoles (LB), swollen mitochondria (arrowhead), and a blunted microvillous border (red arrow). Observe the presence of marked oedematous tissue (*) accompanied by a reduction in alveolar space (AL). Detachment and shedding of deteriorated type I pneumocytes (P1) into the alveolar lumen (AL) are seen. Observe the alveolar macrophage (MQ) exhibiting lamellipodia (bifid arrow) and a kidney-shaped nucleus (n). Observe the eosinophil cell (E**)** exhibiting its distinctive granules (double striped arrow) amidst engorged blood capillaries (RBC). (**G-I**): Excessive collagen accumulation (C) and fibroblast cells (F) are noted. Rarified cells with apoptotic bodies (red arrow) were also observed (TEM, (A, C-G, I) Scale bar = 2 μm and (B&H) Scale bar = 500 nm)

In contrast, group II of the Paraquat study showed a wide variety of ultrastructural degenerative changes, with most type II pneumocytes exhibiting vacuolated lamellar bodies in an irregularly arranged lamellar configuration. Other type II cells’ luminal surfaces showed reduced and irregular blunted microvilli. Furthermore, the majority of pneumocytes exhibited nuclear alterations, including irregular heterochromatic nuclei and enlarged mitochondria. Some regions also showed apoptotic bodies made of type II cells. Prominent fibroblasts and obvious substantial collagen deposition were observed in the inter-alveolar septa. Due to the presence of substantial oedematous tissue, the alveolar lumen was significantly narrowed and, in certain areas, completely obliterated. Type II pneumocytes were more frequent, whereas Type I pneumocytes were less frequent. In addition to RBCs that had extravasated, the inter-alveolar septum also contained macrophages that had lamellipodia and a kidney-shaped nucleus. The eosinophil cell’s characteristic granules were visible within the clogged blood vessels (Fig. 8A-I).

**Fig. 9:**
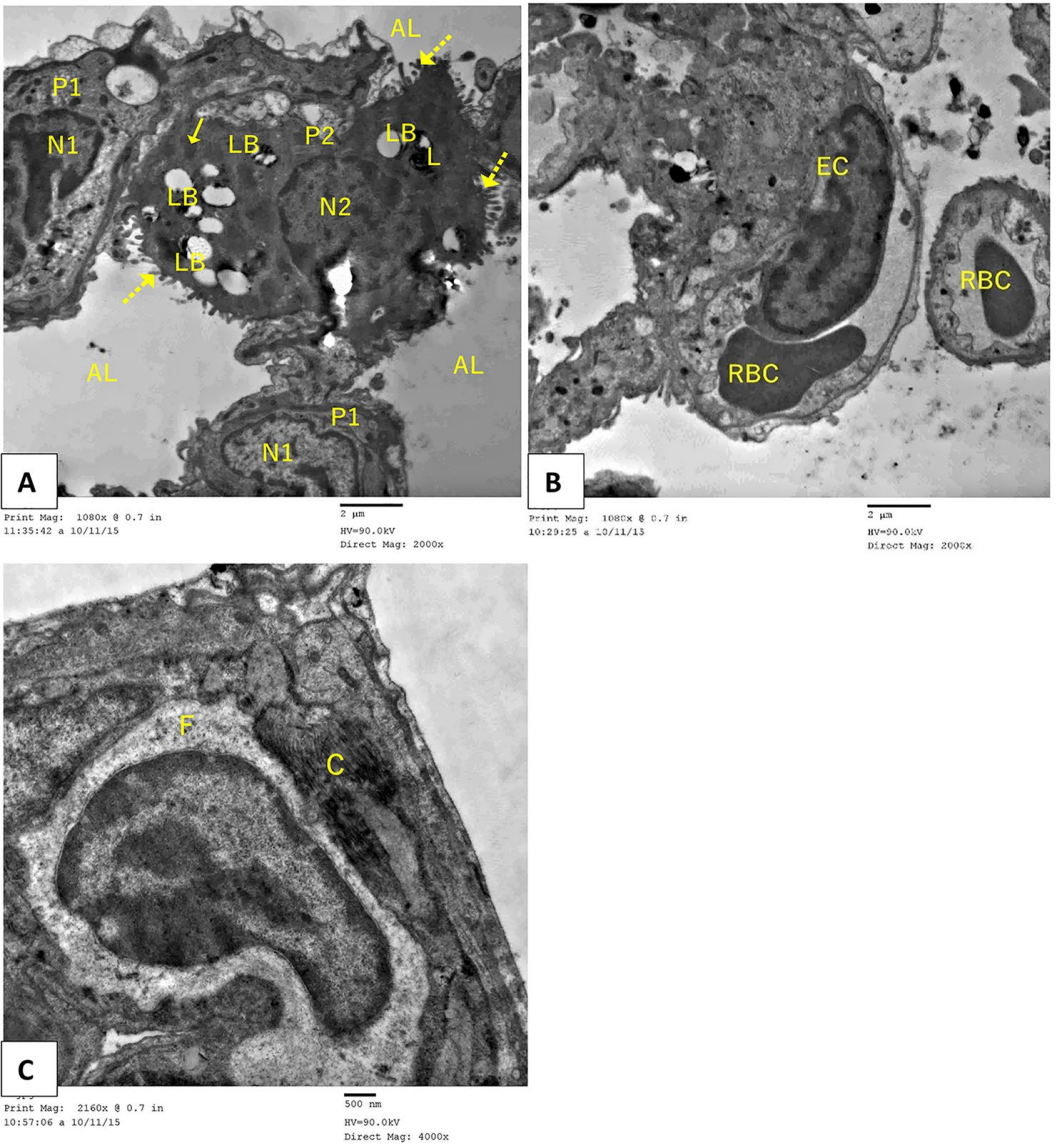
Electron micrographs of an ultrathin section of AT-MSC-treated murine lung tissue showing: (**A**) a type II pneumocyte cell (P2) undergoing exocytosis of lamellar bodies (LB) into the lumen. The nucleus (N2) exhibits euchromatin characteristics. Microvilli (dot arrow) are seen. Lysosomes (L) are observed in the apical region. A patent alveolar lumen (AL) lined by a type I pneumocyte (P1) with an euchromatic nucleus (N1) is seen. (**B**) An endothelial cell (EC) has a normal nucleus and chromatin distribution (TEM, (A-B) Scale bar = 2 μm). (**C**) A Fibroblast (F) with fine collagen fibers is observed (TEM, Scale bar = 500 nm)

Lung ultrastructure showed significant improvement in the group of mice treated with AT-MSCs (Group III), according to examination of lung sections. Normal ultrastructure with euchromatic nuclei was seen in type I and type II pneumocytes. Most of the lamellar bodies were shown by type II pneumocytes. Their apical margin bore noticeable microvilli. The consistent nuclear contour gave the endothelium of blood capillaries a normal appearance. There was fine collagen fiber deposition in the interalveolar septum (Fig. 9A-C).

**Fig. 10:**
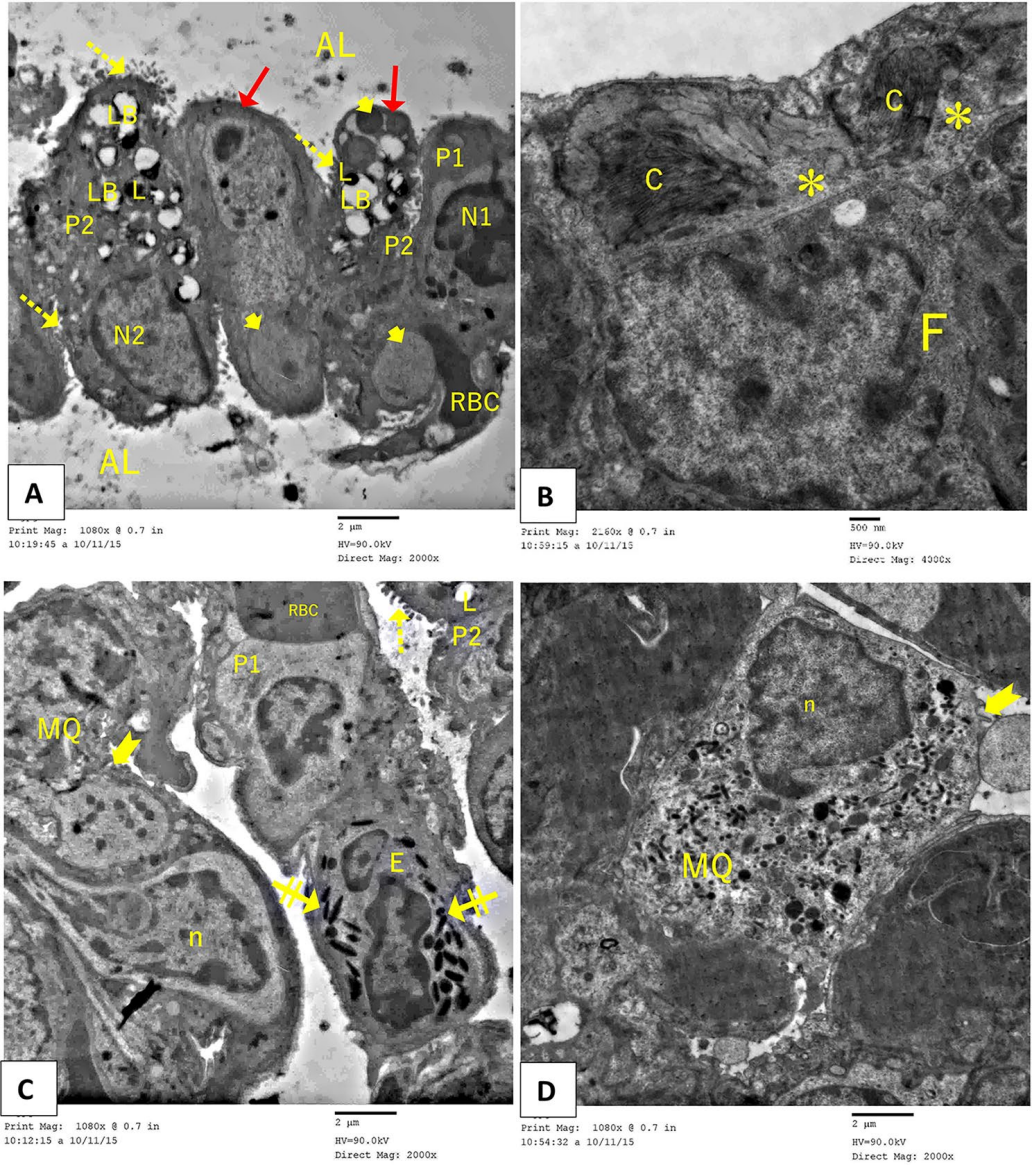
Electron micrographs of an ultrathin section of Rapamycin-treated mice lung tissue showing (**A**): patent alveolar lumina (AL) and type I pneumocyte (P) with an euchromatic nucleus (N1) are observed. A type II pneumocyte (P2) is undergoing exocytosis of lamellar bodies (LB) into the lumen. The nucleus (N2) exhibits euchromatin characteristics. Microvilli (dot arrow) are seen. Lysosomes (L) are observed in the apical region. Some type II pneumocyte (P2) has empty lamellar bodies (LB), swollen mitochondria (arrowhead), and a blunted microvillous border (red arrow). (**B**) Edematous (*) inter-alveolar septum contains a fibroblast (F) with considerable collagen deposition (C). (TEM, Scale bar = 500 nm) (**C**&**D**) An alveolar macrophage (MQ) exhibiting lamellipodia (bifid arrow) and a kidney-shaped nucleus (n), alongside an eosinophil cell (E) characterized by distinct granules (double strick arrow), are detected (TEM, A, C, D: Scale bar = 2 μm, B: Scale bar 500 nm)

The electron microscopy analysis of the Group IV (Rapamycin-treated group) showed that the alveolar tissue was preserved, although there were isolated spots with degenerative cells. Pneumocytes of both types bordered the alveoli, which were patent. Type I pneumocyte and alveolar macrophage were observed. An eosinophil cell with apparently specific granules was observed. A microvillous border, many lamellar bodies, and euchromatic nuclei characterize type II pneumocytes. Sparse lamellar bodies, enlarged mitochondria, and a blunted microvillous border were observed in another degraded II pneumocyte. Within the oedematous inter-alveolar septum, fibroblasts were seen, along with considerable collagen deposition (Fig. 10A-D).

### Assessing the potential for homing and engraftment of AT-MSCs injected systemically in female mice’s lung tissue using Real-Time PCR

**Fig. 11:**
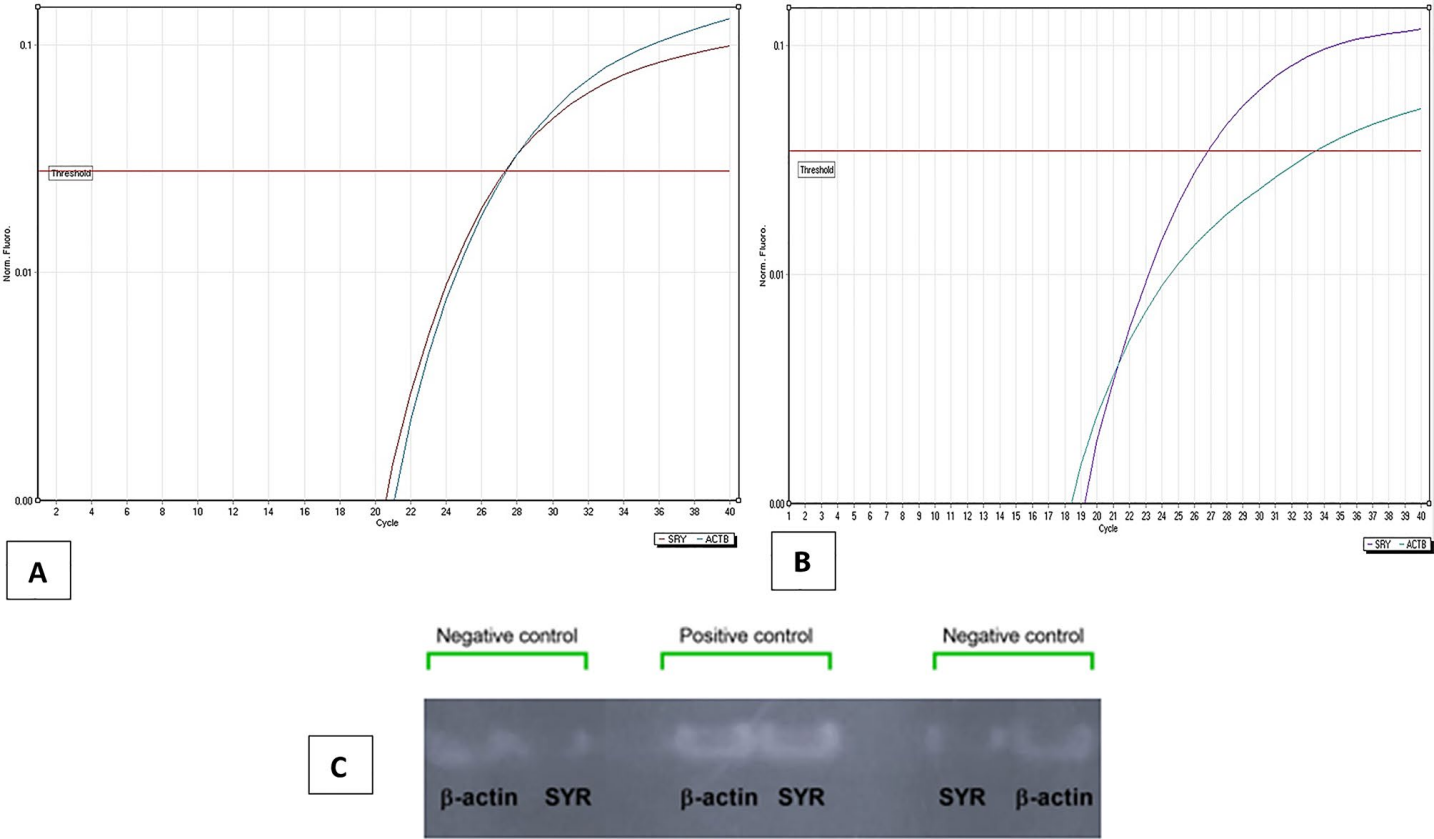
(**A**) Amplification plot illustrating the expression of the SRY gene in female mice (Control group I). (**B**) Amplification plot illustrating the expression of the SRY gene in female mice injected with AT-MSCs from male mice (group III). (**C**) Full-length Agarose gel electrophoresis (1%) illustrates that an amplified SYR gene was identified by a single band (size: 254 bp) and β-actin genes (housekeeper) of the tested samples. The amplified SYR and β-actin genes are identified by a single band (size: 250 bp) in the positive control sample (PQ + AT-MSCs treated group III: female mice injected with male AT-MSCs) sacrificed after 3 days from the injection of AT-MSCs, while the negative control Sample (control group I: female mice received male AT-MSCs) is negative for the SYR gene and positive for the β-actin gene. M: represented the PCR marker

Injecting females with AT-MSCs from males resulted in a significant (*P* < 0.0001) elevation of the Y-chromosome expression levels (a 7-fold change) (1.026 ± 0.24) compared to the negative control group (7.558 ± 0.66) (Figs. 11A& B). In Fig. 11C, the SRY gene PCR products in the lung tissues were identified. The amplified SYR and β-actin genes were identified by a single band (size: 250 bp) in the positive control sample (PQ + AT-MSCs treated group III: female mice injected with male AT-MSCs) sacrificed after 3 days from the injection of AT-MSCs, while the negative control Sample (control group I: female mice received male AT-MSCs) was negative for the SYR gene and positive for the β-actin gene. Full-length blots/gels are presented in Supplementary Fig. 11.

### Biochemical results

**Fig. 12:**
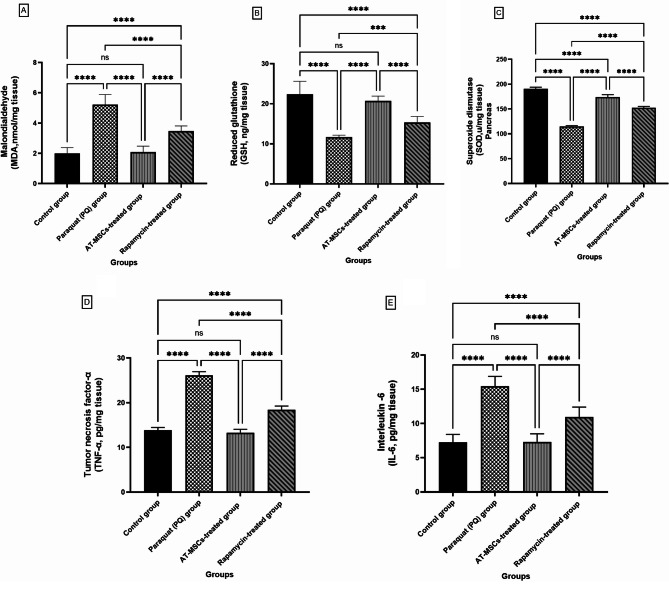
Comparison between the mean values (± SD) of the oxidant–antioxidant status of the mice, three biological replicates per group (*n* = 3). The level of (**A**) malondialdehyde (MDA), (**B**) reduced glutathione (GSH) as well as the activity of (**C**) superoxide dismutase (SOD) and the level of (**D**) Tumor necrosis factor- alpha (TNF-α) and (**E**) Interleukin-6 (IL-6) levels in the lung tissue in all studied groups. The statistical results are shown as mean ± standard deviation (SD). ns: non-significant and **** significant at *P* < 0.0001

The MDA level was considerably higher in groups II and IV as compared to the control group. Among groups II and IV, there was no statistically significant variation in MDA levels. In comparison to the control group, group III treated with AT-MSCs demonstrated a nonsignificant difference in MDA levels; however, when compared to groups II and IV, there was a significant decrease. Groups II and IV demonstrated a marked decline in SOD and GSH activity when contrasted with the control group. Although there were notable variations in their levels between groups II and III. After receiving AT-MSCs (group III), their levels did not differ significantly from those of the control group. However, there was a rather significant rise when compared with groups II and IV (Table 1; Fig. 12A-C).

The level of lung IL-6 and TNF-α levels in female mice administered Paraquat (group II) and Rapamycin treatments (group IV) was significantly upregulated compared with the control group I. Nevertheless, when contrasted with the Paraquat (group II) and rapamycin (group IV) treatments, the AT-MSCs-treated mice showed a substantial reduction in IL-6 and TNF-α levels and activity. Their levels were not significantly different from those in the control group after treatment with AT-MSCs (Table 1; Fig. 12C&D).

## Discussion

One of the herbicides often employed in agricultural productivity nowadays is paraquat, a poisonous bipyridine molecule. On the other hand, even a little PQ consumption might induce deadly lung harm due to its extremely poisonous nature [5, 31]. Toxic effects caused by PQ primarily manifest as acute lung damage and subsequent fibrosis [32, 33]. After ALI treatment, fibrosis is effectively prevented [33, 34]. Although rapamycin can delay the decline of lung function, recent research has shown that it does not extend the longevity of people with IPF. Because of its intricacy and the scarcity of donor organs, IPF continues to be a significant obstacle to the only viable treatment method available at the moment—lung transplantation [35]. So, novel therapeutic approaches and mechanistic studies of PQ poisoning might aid in the development of therapy options. Due to their immunomodulatory, anti-inflammatory, anti-fibrotic, and regenerative characteristics, as well as their low adverse effects in many experimental animal models, MSCs have recently been shown to be significant for cell-based therapy of lung disease [36].

This is why we evaluated the effectiveness of AT-MSCs with that of the gold standard drug, rapamycin, in a mouse model of PQ-induced ALI and lung fibrosis. The purpose of this comparison was to assess the efficacy of stem cells vs. rapamycin as a standard treatment in human clinical trials to prevent severe acute exacerbation. To reduce symptoms of PQ-induced ALI and lung fibrosis, we have demonstrated that systemically given AT-MSCs exhibit anti-inflammatory and anti-fibrotic properties.

While not all MSCs injected into the tail vein will end up in the lung tissue, research has demonstrated that MSCs treated intravenously can reduce pulmonary fibrosis via paracrine processes such as immune response modulation, fibrogenic signaling pathway inhibition, and secretion of anti-inflammatory cytokines [37–39]. In addition, MSCs tend to cluster around injured areas; for example, when it comes to lung injury, a large number of the cells that are injected into the vein temporarily gather in the capillaries of the lungs [38, 39]. This research lends credence to the idea that tail vein injection can effectively alleviate symptoms of lung fibrosis in animal models.

In the present study, a single i.p. injection of 30 mg/kg was administered to mice, and lung tissue samples were obtained after two weeks. Research on PQ poisoning’s mechanism should replicate clinical PQ exposure. For PQ toxicity investigations, researchers have utilized a range of animal species as study models [40]. Researchers frequently choose mice to examine the toxicological process of PQ poisoning [6, 41]. A recent study indicated comparable toxicity and symptoms in humans and animals afflicted with PQ poisoning. Yu et al. indicated that the C57BL/6J mouse strain serves as a significant animal model for investigating hepatic and renal function in cases of PQ poisoning [42]. The lack of chelating agents and particular antidotes makes a 30 mg/kg PQ oral dosage potentially fatal. The lack of chelating agents and particular antidotes makes a 30 mg/kg PQ oral dosage potentially fatal [43, 44]. Cai et al. found that the most prevalent mode of administration, which is consistent with the fact that most PQ poisoning patients are oral, is a single intraperitoneal injection [3].

The clinical progression of PQ-induced pulmonary fibrosis is characterized by three distinct phases: the initial inflammatory phase, the proliferative phase characterized by fibroblast proliferation, and the collagen deposition phase. The inflammatory response is a crucial phase in the progression of pulmonary fibrosis within these stages [6, 45]. Inflammatory cytokines, including TNF-α, IL-1β, IL-6, and TGF-β1, are recognized to play a role in the initiation and progression of pulmonary fibrosis [9, 46, 47]. Inhibition of lung injury healing, promotion of lung cell death, and eventual induction of pulmonary fibrosis can be caused by TGF-β1, IL-1β, IL-6, and TNF-α [9, 48, 49].

The outcomes of our study revealed a distinct inflammatory response in the lung tissue of PQ-treated mice, evidenced by an increase in TNF-α and IL-6 levels. This came in accordance with Zhang et al. [50]. Alveolar collapse, significant leukocyte infiltration, pulmonary edema, and bleeding were among the histopathological alterations induced by PQ exposure in the animals used in the study. In addition, multiple experimental investigations using lung lavage have shown that inflammation is an important mediator of PQ-mediated action. Pulmonary fibrosis may also develop because of uncontrolled inflammation. In ALI caused by PQ, the main inflammatory cells are alveolar dendritic cells, neutrophils, macrophages, and lymphocytes. Additionally, leukocyte infiltration is associated with elevated levels of cell adhesion molecules (ICAM-1), chemokines, and proinflammatory cytokines (TNF-α, IL-1β, and IL-6) [51]. In response to stimuli, activated macrophages, lymphocytes, and epithelial cells release interleukin-6 (IL-6), a key regulator of how inflammatory cells differentiate. In addition to boosting the inflammatory damage response, IL-6 encourages the production of intercellular adhesion molecule-1 and the differentiation and activation of lymphocytes for inflammation. A major systemic inflammatory response can be triggered by IL-6 because it promotes neutrophil respiratory burst and degranulation and stimulates hepatocytes to generate acute-phase proteins [51]. As a result, a decrease in pulmonary inflammation could aid in the healing process following lung damage [48, 49]. Transplantation of AT-MSCs successfully decreased levels of these inflammatory cytokines in the lungs of the mice in the current study. The anti-inflammatory effects of AT-MSCs at 1.0 × 10¹ cells/mouse were comparable to the positive anti-inflammatory effect of rapamycin [52]. Thus, our findings suggest that transplantation of AT-MSCs has an anti-inflammatory impact in mice exposed to PQ.

Research indicates that PQ poisoning induces lung damage by generating oxidative stress via the cyclic oxidation of PQ. This stress subsequently results in multiple occurrences, such as infiltration of inflammatory cells, pulmonary fluid retention, the release of diverse mediators (including cytotoxic, fibrotic, and inflammatory factors), impaired epithelial-mesenchymal transition, enhanced fibroblast proliferation and differentiation, and the accumulation of matrix extracellular components, such as collagen [5]. An essential part of dealing with the massive surge in free radical generation is the work of antioxidant enzymes like SOD and GSH, which may scavenge free radicals to produce more stable components [53]. Additionally, PQ-generated oxygen free radicals cause neutrophil adhesion and infiltration, which in turn produce potent cytokines and chemokines that damage endothelial cells, increase vascular permeability, and cause pulmonary edema [54].

By increasing the activity of antioxidant enzymes like SOD and GSH and by decreasing the rise of MDA in the lungs of mice following PQ intoxication, the current research show that AT-MSCs transplantation mitigates PQ-induced oxidative stress and inflammation. In mice exposed to PQ, our data show that transplantation of AT-MSCs has an antioxidant impact. Animal studies have demonstrated that MSCs can alleviate inflammatory illnesses and other immunological problems, according to multiple sources [55]. Exogenously delivered MSCs have the potential to home to damaged lung tissues and aid in either direct repair (by integrating into the injury site) or lung protection (by transdifferentiating into endothelial cells or pulmonary alveolar epithelial cells). Furthermore, MSCs can inhibit fibrosis and contribute to the immune response to lung injury through their paracrine secretory function [55–58]. Interactions between MSCs and immune cells, as well as the inflammatory milieu, might reduce their engraftment and differentiation capabilities, making it more difficult for them to adequately replace injured tissues [59, 60]. In addition, MSCs reduced the inflow of inflammatory cells and caused proinflammatory cells to undergo apoptosis [61]. Research in animals has shown that MSCs may effectively repair lung damage caused by PQ [51, 62, 63].

Our examination revealed that the lung architecture was severely damaged after intraperitoneal injection of PQ, as confirmed by H&E staining of lung sections. Researchers have utilized animal models of PQ-induced lung fibrosis and damage to study the causes of these conditions and the effects of potential treatments [64–66]. On examination, some alveoli collapsed, while others showed signs of emphysematous dilatation. One possible explanation for the alveolar collapse is that the surfactant is not being broken down quickly enough. It seems that alveolar macrophages couldn’t handle the surfactant produced by hyperplastic pneumocytes type II [67, 68]. Additionally, it was noted that the alveoli and inter-alveolar septa contained homogenous acidophilic material. Similar findings were reported by Hafez [69], who characterized the acidophilic material as plasma exudates generated when the cell wall of blood vessels is injured. The alveolar-capillary barrier may become more permeable, allowing protein-rich fluid to penetrate the septa and for acidophilic exudates to form [70, 71].

The pronounced thickening of inter-alveolar septa observed in group II may be attributed to an accumulation of inflammatory cells, extravasated red blood cells, and clogged capillaries. The activation of macrophages, which produce IL-8, a powerful neutrophil chemotactic agent, and other inflammatory cells led to an increase in inflammatory cellular infiltration in the lungs. As a result, their population in the interstitium and vascular space increases [72].

The blood vessels in the current study had a sclerotic appearance, with a significantly thicker wall. Pulmonary hypertension can develop when the wall of pulmonary arterioles thickens [73]. Red blood cells are extravasated between the alveolar cells and the septa, resulting in congestion of the pulmonary blood vessels. In accordance with these findings, Dinis-Oliveira et al. [74] observed intra-alveolar hemorrhage, the formation of hyaline material, and the aggregation of fibroblasts and macrophages in rats subjected to PQ therapy. The breakdown of red blood cells is the likely cause of the increased number of macrophages with brownish cytoplasmic colour. When exposed to lung toxicants, macrophages quickly accumulated in lung tissue, displaying morphological changes, enlarging, and vacuolization. They also generated more lung TNF-α, a hallmark of cytotoxic and pro-inflammatory macrophages [75].

The present study’s ultrastructural analysis of PQ mice’s lungs corroborated the light and immunohistochemical findings, revealing distinct alterations in the lining of the alveoli. This study elucidated several morphological alterations in the alveolar lining cells, as demonstrated by light and electron microscopy. These changes included a significant reduction in type I alveolar cells, the presence of a few vacuolated cells, and an abundance of cuboidal cells, which were identified as type II alveolar cells by electron microscopy. Accordingly, the alveoli lining was primarily composed of type II alveolar cells. A further study identified analogous changes in the epithelial lining and associated them with the demise of type II alveolar cells, which were subsequently replaced by proliferating type II alveolar cells. They additionally showed that in BLM-induced interstitial pneumonitis, pulmonary epithelial permeability increases because type I cells, which make up most of the alveolar lining cells, are more easily damaged [76]. This is because these cells are specifically targeted by the harmful effects of hyperoxia [77]. In addition, fluid and electrolyte imbalances may be the result of free radical exposure, which disrupts lung biochemical processes [78]. It is worth mentioning that type I pneumocytes exhibited an irregular nucleus and an irregularly indented nucleus, but type II pneumocytes had seemingly expanded mitochondria with destroyed cristae and appeared to have enlarged mitochondria. The elevated levels of ROS and the lipid peroxidation that accompanied them were thought to be responsible for these alterations in nuclear and mitochondrial membranes. Consequently, abnormalities in nuclear membranes and DNA damage occurred [79]. Additionally, ALI group type II pneumocytes exhibited localized cytoplasmic rarefaction and vacuolations. It is commonly believed that the presence of vacuoles indicates deterioration [80]. One possible explanation for these alterations is the oxidative stress-induced deterioration of the cellular organelles. Protein misfolding in the endoplasmic reticulum (ER) also causes ER stress, which manifests as enlarged and compressed tubules and vesicles. In addition, vacuolization would be the result of impaired lipid transport, triglyceride buildup in the cytoplasm or damaged mitochondria, and other alterations [81]. The type II pneumocytes in the ALI group had coalescent empty lamellar bodies. Acute inflammation of the lungs causes problems with surfactant protein synthesis, secretion, and composition, which could explain this [82]. The ALI group exhibited atypical endothelial cells characterized by irregular nuclei and extravasation of red blood cells, potentially attributable to oxidative stress, inflammation, or degradation of the glycocalyx [83].

After AT-MSCs were transplanted, the pathogenic changes improved significantly. According to Masson’s trichrome staining, there was a marked decrease in collagen fiber deposition after AT-MSCs administration. By preventing the buildup of ECM, this finding demonstrates the protective function of AT-MSCs. The anti-fibrotic impact was the primary indicator of AT-MSCs’ protective effects against lung fibrosis, according to these in vivo outcomes. Additionally, two earlier investigations showed that AT-MSCs alleviated IPF and made a substantial contribution to early-stage lung healing [84, 85]. According to Jiang et al., AT-MSCs, when given early on, have the potential to alleviate idiopathic pulmonary fibrosis and make a substantial contribution to lung restoration [84]. As a potential treatment for pulmonary fibrosis caused by repeated bleomycin (BLM) injection, Lee et al. proposed AT-MSCs [85].

A significant elevation in the mRNA expression of the SRY gene, a gene associated with the Y chromosome, was noted in the group administered AT-MSCs. This finding signifies the existence of male-AT-MSCs in the lung tissue derived from male mice. After being introduced to the body systemically, AT-MSCs settled inside the lungs and remained there until the study’s conclusion. It is worth mentioning that AT-MSCs were not found in the control animals’ lungs at the end of the study. This suggests that for AT-MSCs to recruit to the lung, they must first cause tissue injury. Once there, they will respond to inflammatory signals in a specific pattern, protecting the lung from future fibrotic changes [86]. Several studies indicate that MSCs can be directed to wounded regions of the lung, where they facilitate the repair of damaged pulmonary epithelial cells, augment the secretion of surfactants, and mitigate inflammatory reactions [87]. Furthermore, BM-MSC implantation safeguarded animals from PQ-induced acute lung injury and prolonged the survival duration of rats administered PQ [62]. The implantation of MSCs has the potential to physically reduce lung edema, block the release of inflammatory cytokines, and decrease inflammatory responses. Therefore, MSCs have proven to be effective in protecting the lungs from PQ damage. However, it is still mostly unknown what protective mechanisms MSC transplantation mediates [50].

Cell proliferation and apoptosis are recognized as critical pathophysiological processes in ALI, particularly concerning cytoprotection and injury-induced regeneration of lung epithelial cells [88]. Research in the last few years has demonstrated that PQ influences cell death [41, 89, 90]. Research has demonstrated that PQ can trigger extrinsic apoptosis by activating caspase-8, the protein responsible for starting the extrinsic apoptotic pathway [91]. A tumor suppressor gene that has received a lot of attention and study is the P53 gene. It is common to think of p53 as a guard gene because of its important roles in normal cell proliferation, tumor inhibition, and cell cycle regulation [92, 93]. In a transcription-dependent or -independent way, P53 can be adjusted to stress when it is activated by a variety of stressors [94]. Extensive studies have shown that p53 is essential for the control of lung fibrosis and cancer. The link between p53 and fibrosis of the lungs has been the subject of a few investigations [95]. Researchers established a mouse model of lung fibrosis by administering BLM intratracheally in a single instance. The findings indicated that mice with pulmonary fibrosis exhibited markedly increased levels of the p53 protein and dramatically enhanced apoptosis of type II alveolar epithelial cells in their lung tissues. Conversely, pulmonary fibrosis and death of type II alveolar epithelial cells were markedly diminished following the knockout of the p53 protein. This suggests that the p53 protein likely accelerated the formation and progression of pulmonary fibrosis by inducing apoptosis in alveolar epithelial cells [96]. Additional studies have utilized intratracheal administration of BLM in both wild-type and p53-deficient mice to develop models of pulmonary fibrosis. In comparison to wild-type mice, p53-deficient mice exhibited significantly reduced lung tissue damage and diminished collagen deposition, as indicated by these data [97]. Inhibiting p53 expression may halt the development of pulmonary fibrosis, according to these results. Compared to PQ-exposed mice that did not receive AT-MSCs or mice treated with Rapamycin, the present investigation found that PQ-exposed mice treated with AT-MSCs had significantly lower expression levels of the IHC stain of P53. These results show that after PQ intoxication, transplantation of AT-MSCs reduces PQ-induced apoptosis in mice’s lungs by increasing IHC staining of P53. According to our findings, transplanting AT-MSCs into PQ-exposed mice has an antiapoptotic impact.

Inflammation, angiogenesis, and fibroblast activity could all be effectively suppressed by Rapamycin [98, 99]. Several studies found that Rapamycin slowed the progression of pulmonary function [100, 101]. Rapamycin alleviates alveolitis and pulmonary fibrosis in the BLM-induced pulmonary fibrosis rat model by reducing the production of matrix metalloproteinase (MMP)-9 and tissue inhibitors of MMP-1 in lung tissue [102]. It has been demonstrated by Tai et al. [16] that rapamycin alleviates PQ-induced lung fibrosis and decreases the expression levels of collagen (Col)-I, Col-III, MMP-2, and MMP-9. On the other hand, Veret et al. [15] investigated the possible advantages of rapamycin and AT-MSCs on chondrocytes obtained from osteoarthritis patients. When comparing the phenotypic of osteoarthritis chondrocytes treated with AT-MSCs and rapamycin alone to those treated with the combination, they found that the synergistic restorative benefits were much stronger. They verified that in vitro isolated chondrocytes from patients with osteoarthritis showed reduced expression of senescence/fibrotic markers when treated with rapamycin. As a result, the combination of rapamycin with AT-MSCs is a promising option for protecting therapeutic cells from the unintended consequences of joint senescence [15].

## Electronic supplementary material

Below is the link to the electronic supplementary material.

Supplementary Material 1

Supplementary Material 2

## Data Availability

The data used to support the findings of this study are included in the article.

## Declarations

### Artificial intelligence (AI)

The authors declare that they have not used AI-generated work in this manuscript.
