# Supplementary figures and images for: Comparative study of adipose tissue derived mesenchymal stem cells with rapamycin on paraquat-induced acute lung injury and pulmonary fibrosis in a mouse model: histological and biochemical study
Source: Stem Cell Res Ther. 2025 Jul 15;16:377. doi: 10.1186/s13287-025-04498-w (PMC12265330; doi:10.1186/s13287-025-04498-w)

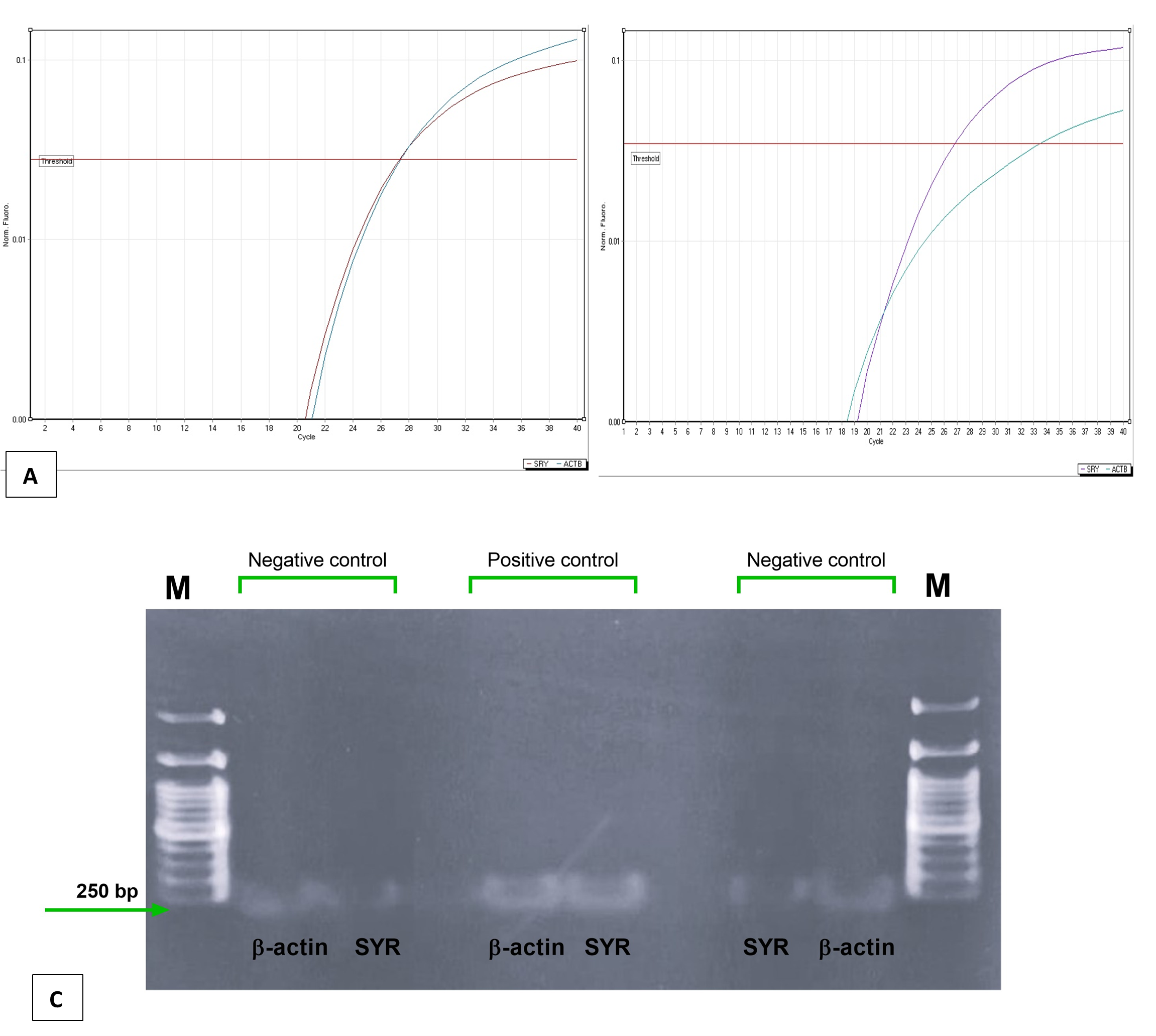

Supplement: Supplementary file 1 — Supplementary Material 1 [file 13287_2025_4498_MOESM1_ESM.tif]

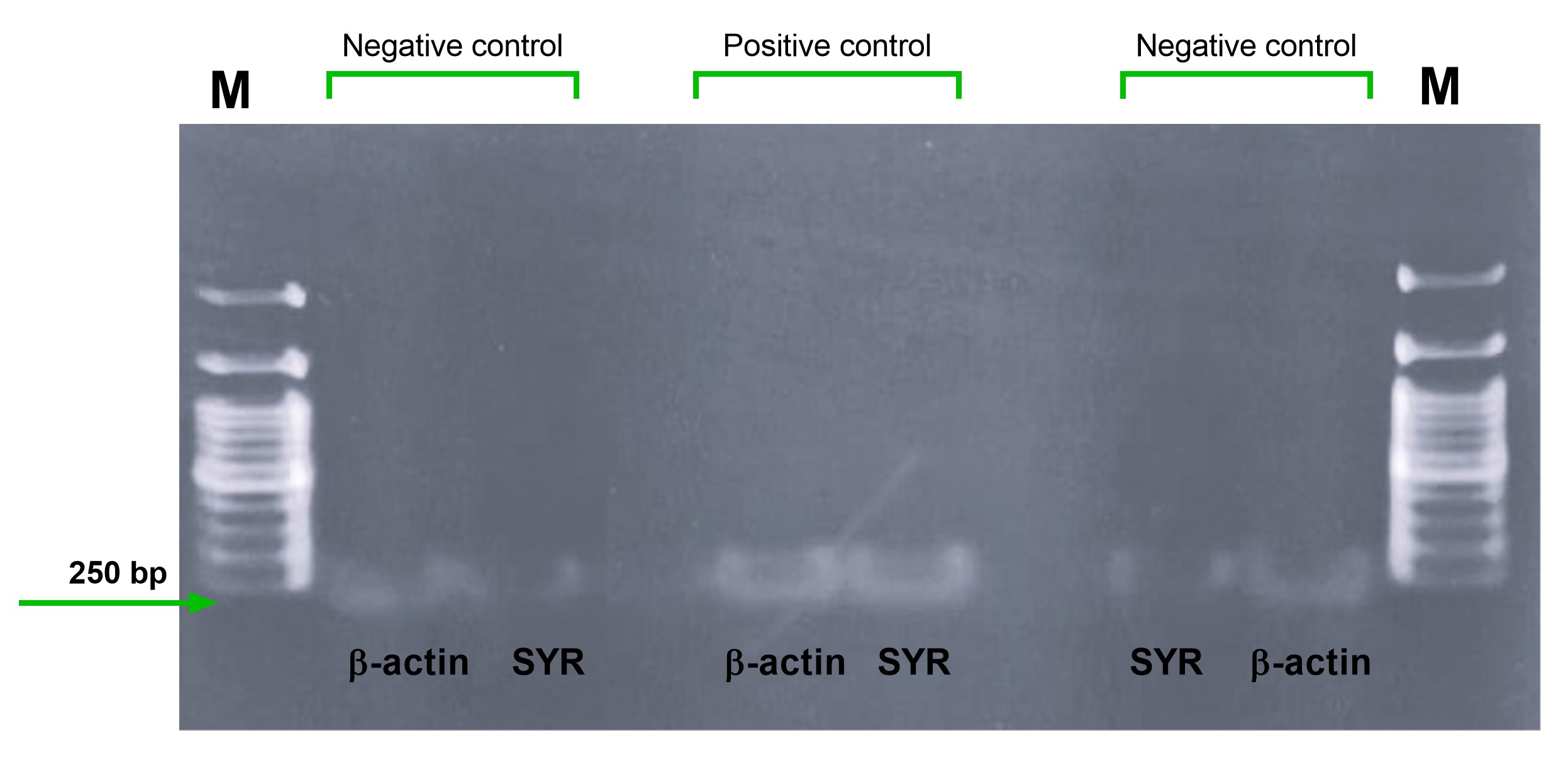

Supplement: Supplementary file 2 — Supplementary Material 2 [file 13287_2025_4498_MOESM2_ESM.jpg]
